# IA-Lab: A MATLAB framework for efficient microscopy image analysis development, applied to quantifying intracellular transport of internalized peptide-drug conjugate

**DOI:** 10.1371/journal.pone.0220627

**Published:** 2019-08-01

**Authors:** Adam M. Corrigan, Johan Karlsson, Jan Wildenhain, Laurent Knerr, Maria Ölwegård-Halvarsson, Maria Karlsson, Svenja Lünse, Yinhai Wang

**Affiliations:** 1 Discovery Sciences, R&D, AstraZeneca, Cambridge, United Kingdom; 2 Discovery Sciences, R&D, AstraZeneca, Gothenburg, Sweden; 3 Research and Early Development, Cardiovascular, Renal and Metabolism, BioPharmaceuticals R&D, AstraZeneca, Gothenburg, Sweden; 4 Research and Early Development, Respiratory, Inflammation and Autoimmune, BioPharmaceuticals R&D, AstraZeneca, Gothenburg, Sweden; University of California Berkeley, UNITED STATES

## Abstract

This work presents a MATLAB-based software package for high-throughput microscopy image analysis development, making such development more accessible for a large user community. The toolbox provides a GUI and a number of analysis workflows, and can serve as a general framework designed to allow for easy extension. For a new application, only a minor part of the object-oriented code needs to be replaced by new components, making development efficient. This makes it possible to quickly develop solutions for analysis not available in existing tools. We show its use in making a tool for quantifying intracellular transport of internalized peptide-drug conjugates.

The code is freely available as open source on GitHub (https://github.com/amcorrigan/ia-lab)

## Introduction

High-throughput microscopy image analysis is an area of increasing importance for biomedical research in academia and the pharmaceutical industry, with experiments and subsequent image analytics becoming ever more complex. Peptide-drug conjugates (PDCs) represent an important class of therapeutic agents in the context of targeted drug delivery and new therapeutic modalities [1, 2], and several approaches using synthetic ligands have recently entered the clinic. PDCs aim to improve the treatment efficacy of a drug through targeted delivery and release in specific cells or tissues [3, 4]. This is achieved by combining one or more drug molecules with a potent and selective homing peptide designed to target specific surface receptors, that potentially allow internalization of the conjugate, through structural compatibility of the peptide [5, 6].

Quantification of intracellular transport is of importance for PDC characterization and chemical optimization within pharmaceutical research, in particular with respect to linker development. In order to characterize PDCs and optimize their chemistry, it is important to understand the intracellular transport mechanisms, which can be observed through fluorescence microscopy. This requires the construction of an image analysis workflow which robustly and quantitatively captures the phenotypic difference between candidate formulations, which is a very common task in high content imaging.

Off-the-shelf tools (e.g., Columbus, MetaXpress, Imaris, Definiens) are often used for everyday image analysis needs, but for novel experimental designs they have limited adaptability. There are several open source platforms available [7] for development of image analysis applications based on implementations in languages such as Python, C/C++, R and Java, and efforts which aim to collect and standardize analysis procedures across the research community [1, 2]. Popular tools include BioImageXD [8], CellProfiler [9], Fiji [10], ImageJ [11], Icy [12] and HCS analyzer [13]. These tools provide the user with the possibility to design a number of standard image analysis workflows without any coding by using existing building blocks with adjustable parameters. However, when what is needed for the application at hand is not available using these building blocks, significant programming effort is required to implement new workflows.

MATLAB is a widely used computing environment, and there are many tools available for microscopy image analysis, such as CellSegm [14], Cellstat [15], Celltracer [16] Celltracker [17]. The Microscopy Image Browser (MIB) is an open source MATLAB tool for bespoke analysis of high-dimensional large image data [18]. CellAnimation [19] is an open source tool for cell tracking where workflows can be built from components. However, a generally applicable, open source available, MATLAB-based framework for high-throughput microscopy image analysis tool development is lacking.

This work introduces a MATLAB-based framework for efficient development of customized high-throughput microscopy image analysis with support for well-plate-based batch analysis, and designed for efficient extension. We show that for the novel PDC imaging assay, existing tools are not able to robustly quantify the phenotypic effect observed. We therefore use our MATLAB framework to build and apply a workflow for the sensitive quantification of intracellular transport of internalized (PDCs). We further demonstrate the utility of our software with applications in 3D spheroid quantification and incorporation of manual supervision in pellet analysis.

The software code is freely available on GitHub (https://github.com/amcorrigan/ia-lab). To enable testing and reproducibility of the results, image data and associated experimental metadata are available on the Dryad Digital Repository (https://clicktime.symantec.com/3292iekDrGXtDhiU6vqG1r36H2?u=https%3A%2F%2Fdatadryad.org%2Freview%3Fdoi%3Ddoi%3A10.5061%2Fdryad.s3v72hb).

## Design and implementation

### ImageAnalytics-Lab (IA-Lab)

Our tool ImageAnalytics-Lab (IA-Lab) supports a graphical user interface (GUI) for navigating plate data and images and for tuning and running analysis workflows. The end user can use applications developed in the IA-lab framework without any programming experience (see S2 File for detailed walkthrough). The framework can also be used via the MATLAB command line or scripting, e.g. to run analyses in parallelized batch runs on a computer cluster. IA-Lab analysis workflows are built by putting different modules together, typically import, segmentation, feature measurement and data export components. The framework includes ready to use modules for each stage of the workflow. For example, import modules exist for a range of imaging formats, segmentation modules include several common cases like cell segmentation with and without nuclei markers, and export modules to standard formats like comma- and tab-separated value are ready to be used. All components are designed to be easily extended or replaced, thereby making development efficient. IA-Lab has an object-oriented design with classes for its core functionalities and image data formats.

Using this modular approach, the coding required for new analyses is minimal, since a new segmentation component, for example, can interact with the existing import, visualization, parameter adjustment, measurement and export modules to quickly produce a functional tool. These three factors; MATLAB, modular build, and object-oriented design, facilitate highly efficient development of solutions for new types of image analysis workflows.

Behind the user interface, plug and play functionality is enabled by defining a number of generic types of data–image data, label matrices (for segmentation results) and structures to store general measurement data. Modules are then implemented as MATLAB classes with defined inputs and outputs for each type of module. For instance, a segmentation module returns one or more label matrices, and takes as inputs image data and optionally existing label matrix data. Modules also define which parameters of the contained algorithm can be varied, thus facilitating supervised calibration of parameters in a generic way–when writing a new module, by defining adjustable parameters the module can be used in IA-Lab’s manual calibration interface, without any additional coding. Workflows are built up by linking the outputs from one module as the inputs of another, and can be thought of as a directed acyclic graph. A benefit of this approach is that if the output of a specific intermediate module is required, for instance during manual tuning of parameters, only the modules required to generate the output can be run, rather than running the whole workflow, saving considerable time for complex workflows. The architecture of IA-Lab is shown in S2 File, illustrating how modules interact with the GUI elements and how to implement a new module. A full description of currently available modules is also provided in S3 File.

Whilst a new assay may require a novel piece of image analysis or a specific measurement to be written, the final output will often be a table of numbers, either per cell, per image or some other method of aggregation. By storing the measurement data in a standardized way as a MATLAB struct, we can use generic export modules to aggregate and write the results in standard formats for downstream visualization or analysis, for instance in Excel, R or Genedata Screener. This approach obviates the need to output results in a bespoke manner for each assay, which can be time consuming and error prone. The tool comes with a number of ready-to-use implemented workflows as well as import tools supporting a wide range of formats. The PDC internalization assay was acquired using a Yokogawa CV7000 confocal microscope. Acquisition metadata, stored in Yokogawa XML files alongside the image files, is read by the Yokogawa Parser module to provide selection of specific wells, fields or z-planes. Within IA-Lab, we have also created parser modules for ImageXpress and Cellomics (via the Bio-formats library [20]) plate-based microscopy, as well as a general parser for nested folders of images. IA-Lab is actively used and developed within AstraZeneca, and therefore compatibility with future versions of MATLAB will be maintained.

### Intracellular transport

In order to quantify intracellular transport of PDCs, development of a custom analysis workflow was needed. A key feature is that the focus for the payload aggregation can be anywhere within the cell, and it can often be in, for example, a subcellular compartment that is not fluorescently marked in the images. This means that generic algorithms, existing in available tools, that are based on aggregation to the cell centroid, distance from cell boundary, or aggregation to some fluorescently marked compartment, do not give optimal results. As a reference, a CellProfiler pipeline was implemented, involving cell segmentation, definition of a series of bands further and further away from the cell membrane, measurement of thresholded PDC signal in those bands, and summarizing as a dispersion measurement from 1 to 4 with 1 representing the signal being mostly far inside the cell and 4 representing the signal being mostly close to the membrane. Since this does not capture the way the PDC aggregates, there was a need for a custom-made workflow.

Using the IA-Lab framework, we used existing modules for GUI, file browsing, cell segmentation, and export of resulting measurements. Only the code for the specific quantification of aggregation of fluorescence signal to sub-regions within cells had to be written as a new module, and even for that module, a large part of the code was reused from a component for another type of quantification of cell content. We used the tool to quantify intracellular aggregation patterns of four different formulations in living cells.

#### Dispersion algorithm for intracellular transport measurement

The algorithm has the following steps, incorporated into a single measurement module.

1. Input is segmented cells (segmented using an existing module in the framework) and corresponding images with the signal that we want to measure aggregation for (typically confocal fluorescence microscopy images with spots showing internalized payload of some sort).
2. Within each cell, automatically threshold the signal to get a binary mask of spots.
3. Calculate the centroid of that mask. This gives the focus point around which the spots are aggregating. This step is critical, since the aggregation is not to the center of the cell or to the nucleus.
4. For each point in the spot mask, calculate the distance to the centroid.
5. Normalize those distances for cell size, by dividing by the diameter of the cell, as defined by the maximum distance between two points in the cell.
6. Finally calculate the mean of the normalized distances from spots to centroid. This gives, for each cell, a value between 0 and 1, with low values corresponding to all spots aggregated into a small region within the cell, and large values corresponding to spots very far from each other. We refer to this value as the dispersion.

## Results and discussion

### Intracellular transport application

**Fig 1:**
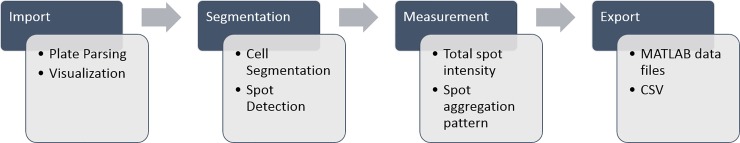
Workflow components. Putting components together to form an analysis workflow. Each major part of the workflow can contain several subcomponents. In this case, only the “Spot aggregation pattern” subcomponent had to be built and the rest of workflow uses available components.

The new module was used in a workflow together with existing modules, as seen in Fig 1, to quantify intracellular aggregation patterns of four different PDC formulations: Alexa-488 labeled human transferrin (488-Tf, T13342 Invitrogen), a glucagon-like protein-1 (GLP-1) receptor agonist labeled at the C terminus with BODIPY FL, a stable peptide-small molecule conjugate of the same GLP1 receptor agonist and Bodipy FL labeled small molecule cargo, a cleavable peptide-small molecule conjugate of the same GLP1 receptor agonist and a BODIPY FL labeled small molecule cargo. The conjugations are using chemical functionalization of the C terminal amino acid on the peptide and a linker to the small molecule which is for the stable conjugate designed to be stable inside cells and not release the labelled small molecule upon internalization. For the cleavable conjugate the linker is designed to be labile in the intracellular milieu and thus trigger the release of the labelled small molecule upon internalization (see S5 File for formulation schemes).

**Fig 2:**
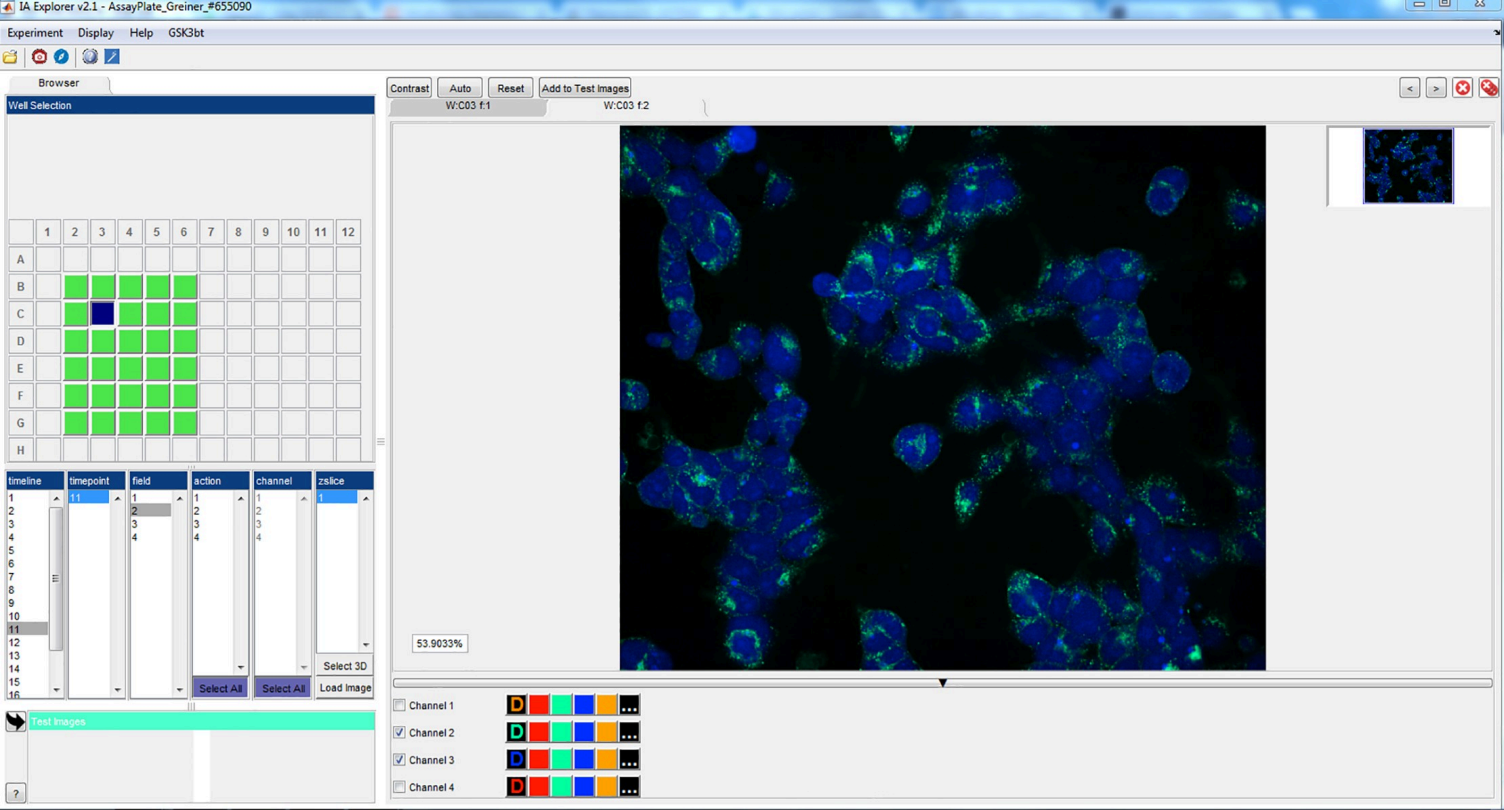
Browsing images using IA-lab. An example of the main browsing view. To the left wells on a plate can be selected, as well as exactly which image from that well is to be viewed. To the right the image is viewed and functionality like zooming, contrast, and turning image channels off is easily available.

**Fig 3:**
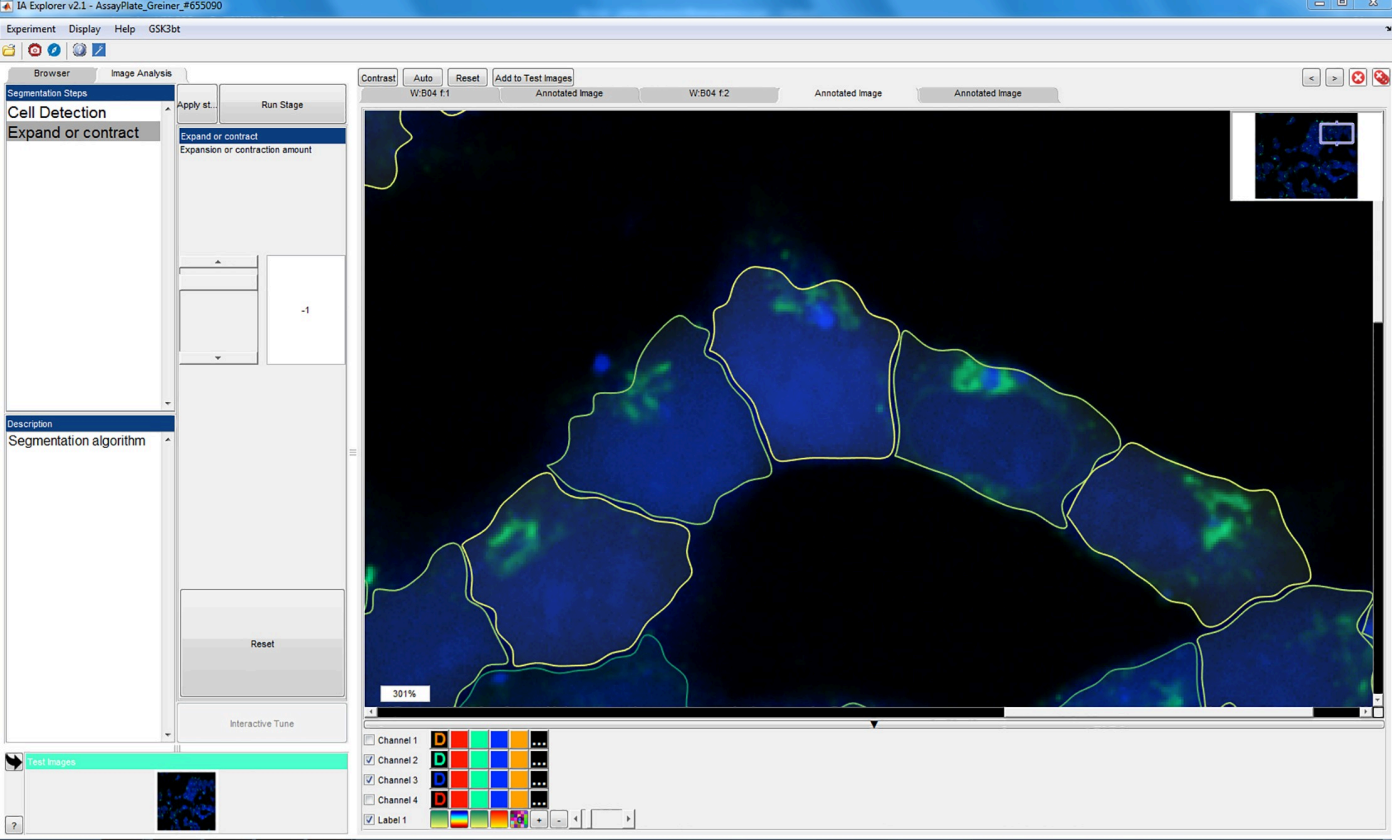
Parameter tuning in IA-lab. In this view, parameters for an analysis workflow can be interactively tuned using selected example images, before running a batch analysis of the entire plate.

HEK cells overexpressing GLP-1 receptor and thus internalizing the different formulations, were imaged live using automated confocal fluorescence microscopy. The image data was processed with IA-lab. Fig 2 shows browsing the images from the well-plate and Fig 3 shows interactive parameter tuning for the workflow. The interactive parameter tuning is done using selected images, and the same parameter values are then applied when running the analysis on all images from the full plate. A benefit of our approach is that when adjusting a parameter, the effect can be simultaneously observed on several images across the range of assay conditions or treatments.

**Fig 4:**
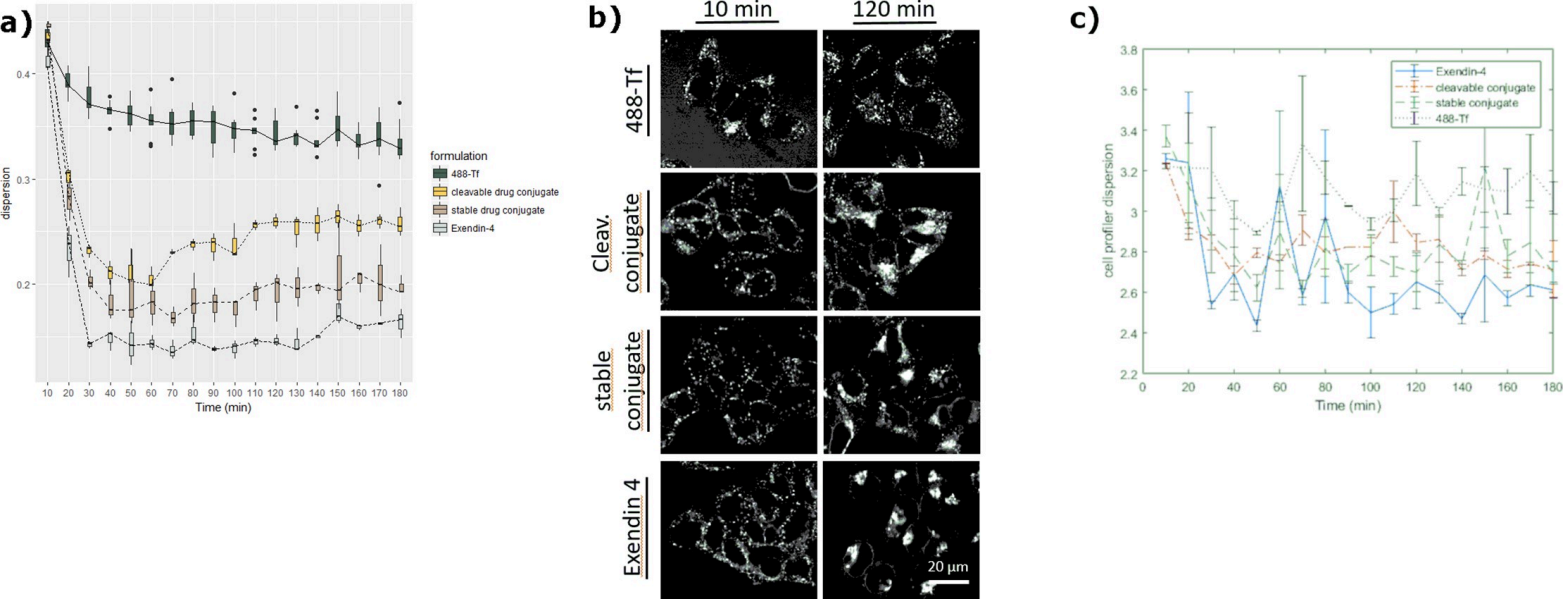
Significant separation of distinct particle dispersion levels using live cell imaging. HEK cells overexpressing GLP-1 receptor, internalizing 488-Tf, GLP-1 -BODIPY FL, two conjugates containing BODIPY FL-labeled small molecule payload (stable versus cleavable linker system), were imaged live and Tiff files were processed with IA-lab. (a) IA-Lab quantification of dispersion plotted as time series, curves show four significantly distinct levels of intracellular dispersion, allowing separation in transport behavior (ANOVA p-value: for separation between 488-Tf and cleavable conjugate p<2×10^−16^ and for separation between cleavable and stable conjugate p = 0.003). (b) Fluorescent-labeled conjugate formulations at times indicated after internalization, changing from similar appearing aggregation patterns at 10 min to significantly different aggregation patterns at 120 min. (c) CellProfiler quantification of dispersion does not capture the way formulations aggregate and results are less clear.

Measurements were exported and plotted as time series curves showing four significantly distinct levels of intracellular dispersion, as seen in Fig 4A. Uptake of 488-Tf was assayed in order to visualize clathrin-dependent endocytosis and recycling endosomal pathways. All four formulations resulted in a similar dispersion value between 0.4–0.45 ten minutes after internalization, showing vesicular occupancy (Fig 4A and 4B, 10 min). While 488-Tf remains in clathrin-dependent endocytosis and recycling vesicles throughout the experiment time of 180 minutes leading to a dispersion value of ~0.35, GLP-1 -BODIPY FL was transported through the cell, clearly in a different manner. Starting from 10 min after internalization and leading to full aggregation over time, GLP-1 -BODIPY FL resulted in the lowest level of dispersion with values of ~0.15 starting from 30 min after internalization. The two peptide-small molecule conjugates containing BODIPY FL-labeled small molecule payload were transported through the cell significantly differently from 488-Tf as well, starting from 10 minutes after internalization, generating final levels of dispersion of ~0.2 for the stable linker versus ~0.25 for the cleavable linker system. The two conjugates were diverging in dispersion levels starting from 60 min after internalization. Comparing their levels of dispersion from 120 minutes onwards using repeated measurements ANOVA gives a highly significant difference (p = 0.003). Here, linker cleavage can be an explanation for the divergence in dispersion levels between the two GLP-small molecule conjugates. The difference between the cleavable conjugate (~0.25) and 488-Tf (~0.35) is also significant (p <2×10^−16^) in a similar test. The difference between the stable conjugate and GLP1-BODIPY FL is also clearly seen in the plots, but it is not statistically significant. Overall, the new module allowed robust and sensitive quantification of intracellular aggregation patterns showing statistically significant differences in particle transport e.g. cleavable versus stable peptide-small molecule conjugates containing BODIPY FL-labeled small molecule payload, enabling PDC characterization and chemical optimization, in particular with respect to linker development. This can be compared with the quantification possible without building a custom workflow in IA-Lab and instead using available standard components in e.g. CellProfiler. Fig 4C shows the result of the CellProfiler pipeline described above. Since the available standard components cannot accurately capture the way the formulations aggregate, the results are less clear and robust conclusions cannot be drawn.

### Further workflows

To illustrate how custom functionality can be flexibly implemented by inheriting from and extending core modules, we describe a number of additional workflows, each integrating a bespoke measurement into a standardized workflow.

**Fig 5:**
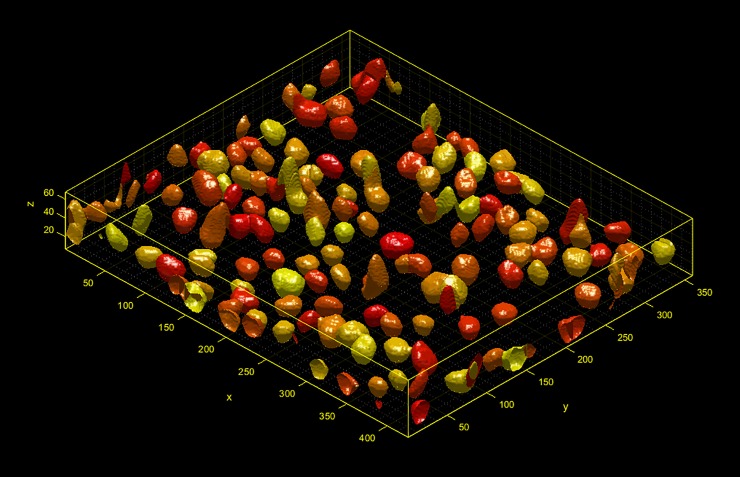
3D cell segmentation. Components are available for segmentation not only in 2D but also in 3D as illustrated here with segmentation of nuclei in a cell layer.

Fig 5 shows segmentation of cells in 3D image stacks. To illustrate 3D analysis in a cellular context, we developed a workflow to measure the nuclear and cell morphology from a stack of fluorescent images. We constructed modules for segmentation of nuclei and cytoplasm in 3D, and for the measurement of three-dimensional morphology. In both cases, although the image processing steps required new modules to be created, the loading of images and export of results were handled by existing components of the core framework.

**Fig 6:**
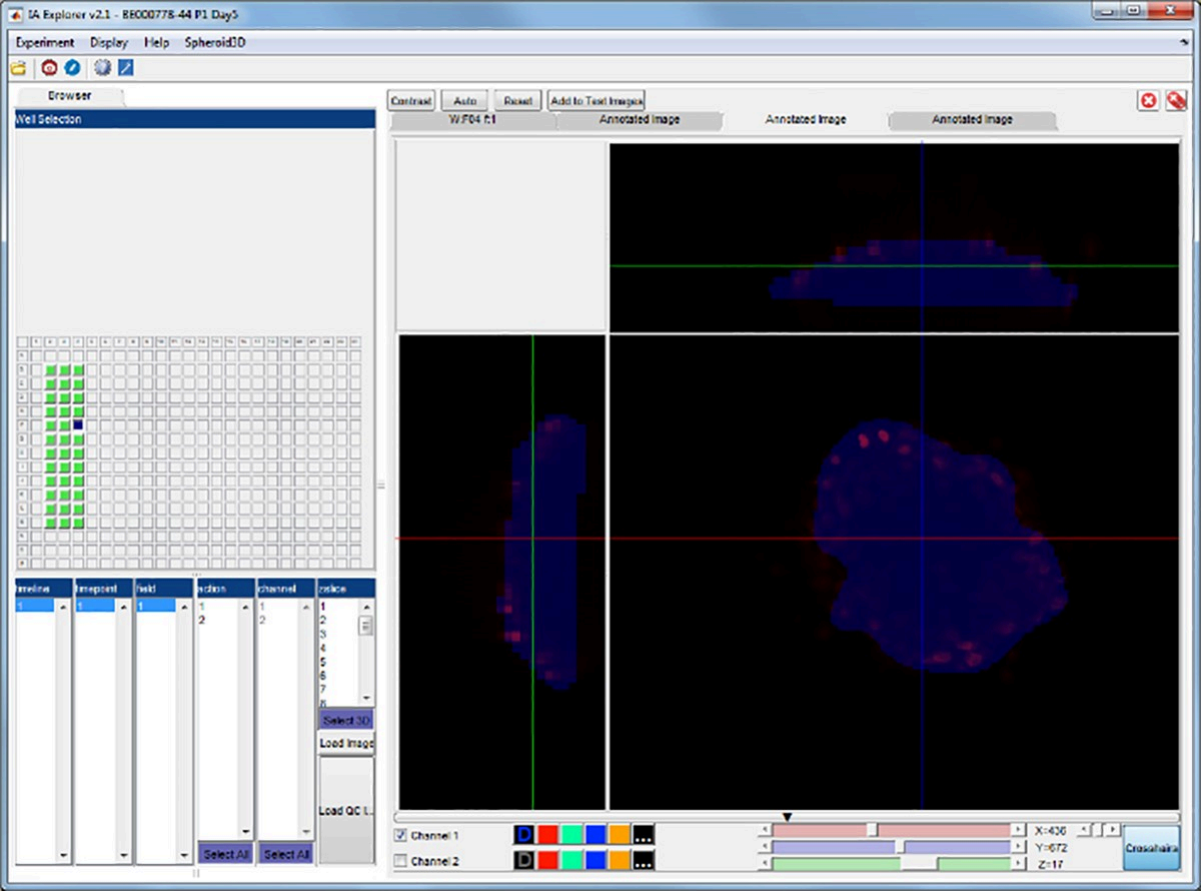
3D spheroids. Quantification of 3D spheroids using available modules together with custom measurement modules.

Fig 6 shows a screenshot from a workflow for the volume and surface area measurement of 3D spheroids. The optical density of the spheroids means that the fluorescence intensity is strongly attenuated beneath the surface of the spheroid; to reconstruct the spheroid volume from a ‘shell’ of intensity, we designed a segmentation method using active contours, and implemented a custom measurement module to calculate properties such as spheroid volume and surface area.

An additional requirement for many medium-throughput assays is efficient manual supervision of results, for cases where absolute accuracy is prioritized above overall speed of analysis. We show an example of this use case, the quantification of the color of rat faecal pellets as an indication of nausea and emesis. The capture of pellet color using photography is influence by light variations and undesirable shadowing. Besides the challenge of touching/overlapping objects (pellets), this creates extra difficulties in the image segmentation and measurements of each pellet. To address this, we created a plugin module with a full GUI to perform supervised post-analysis corrections. This allows scientists to manually fix over-segmentations interactively. We were able to reuse the majority of modules for image loading and visualization while allowing full flexibility in workflow execution. This workflow and additional details about the aforementioned workflows can be found in the S4 File.

Absolute speed performance is not the primary goal of our framework; nevertheless, the workflows presented above can be run on a standard laptop (tested on Intel Core i5-5300 2.3GHz, 8GB RAM). Although the speeds are dependent on the specific analysis, typical 2D analysis workflows run at approximately 10s per image for full resolution images of dimensions 2560×2160 pixels, and 3D workflows around 60s per image. Using MATLAB’s built-in parallel processing options increases the speed by a factor of 2–3 using 4 cores, while deploying to a high-performance computing environment is straightforward and allows an increase in throughput of more than an order of magnitude.

## Conclusion

In summary, this work introduces a MATLAB-based tool and framework for efficient development of customized high-throughput microscopy image analysis. The tool including source code is freely available under the LGPLv3 Open source license on GitHub (https://github.com/amcorrigan/ia-lab). The framework is used to implement novel and sensitive quantification of intracellular transport of internalized PDCs. The high sensitivity of the utilized quantification method is proven by its ability to visualize significant differences in transport behavior for four different PDC formulations, especially two peptide-small molecule conjugates containing BODIPY FL-labeled small molecule payload with different linker stability. Quantification of intracellular transport using our method is supporting PDC characterization, chemical optimization and drug discovery project chemistry decision making, in particular with respect to linker development. Methods to quantify intracellular transport are of importance not only for PDC characterization and chemical optimization but also for other delivery modalities such as antibody-drug conjugates (ADCs) or other drug delivery systems including nanoparticles.

A current shift is occuring, from classical small molecule approaches to new therapeutic modalities such as peptides, antisense oligonucleotides or the use of small molecules in novel ways, for example, targeted or in form of proteolysis targeting chimeric molecules (PROTACs). While these developments will potentially allow a far broader biological space to be targeted [21] they also bring new challenges with regards to understanding the mode of actions and properties of these new modalities at cellular and subcellular levels. This is of high interest for pharmaceutical industry and academia, and as new modalities and complex assays continue to be developed, the importance of maximizing the insight gained using custom image analytics will continue to grow.

## Supporting information

S1 File Dependencies. List of code dependencies.
(DOCX)

S2 File IA-Lab architecture. Class diagram and design of IA-Lab modules.
(DOCX)

S3 File Available workflow modules. List of currently available workflow modules with brief description.
(DOCX)

S4 File Additional analysis workflows. Further examples of different types of workflows.
(DOCX)

S5 File Supplementary Schemes formulations. Formulations tested in the internalization assay.
(PPTX)
