# Supplementary material for: IA-Lab: A MATLAB framework for efficient microscopy image analysis development, applied to quantifying intracellular transport of internalized peptide-drug conjugate
Source: PLoS One. 2019 Aug 1;14(8):e0220627. doi: 10.1371/journal.pone.0220627 (PMC6675096; doi:10.1371/journal.pone.0220627)
Supplement: S1 File — List of code dependencies. (DOCX) [file pone.0220627.s001.docx]

Dependencies

# MATLAB TOOLBOXES

IA-lab is built using Matlab R2014b and uses the following toolboxes:

## MATHWORKS MATLAB TOOLBOXES

Image Processing Toolbox

Computer Vision System Toolbox

Parallel Computing Toolbox

Statistics Toolbox

## EXTERNAL TOOLBOXES AND FUNCTIONS

• Bio-formats library, http://www.openmicroscopy.org/site/products/bio-formats.

• imoverlay, v1.3 - Matlab file exchange.

• colorGradient: generate custom linear colormaps, v1.0 - Matlab file exchange.

• GUI Layout Toolbox, v2.3.1 - Matlab file exchange.

• Add Text To Image, v1.2 - Matlab file exchange.

• Multi ROI/Mask Editor, v1.0 - Matlab file exchange.

• SC - powerful image rendering, v1.10 - Matlab file exchange.

• JSONlab: a toolbox to encode/decode JSON files, v1.5 - Matlab file exchange.

• DIPUM Toolbox, v1.1.4, Gonzalez, R. C., Richard E. Woods, and Steven L. Eddins. "Digital Image Processing using Matlab." Gatesmark Publishing; 2nd edition (2009)
