# Supplementary material for: IA-Lab: A MATLAB framework for efficient microscopy image analysis development, applied to quantifying intracellular transport of internalized peptide-drug conjugate
Source: PLoS One. 2019 Aug 1;14(8):e0220627. doi: 10.1371/journal.pone.0220627 (PMC6675096; doi:10.1371/journal.pone.0220627)
Supplement: S2 File — Class diagram and design of IA-Lab modules. (DOCX) [file pone.0220627.s002.docx]

# High-Level Class Hierarchy

#
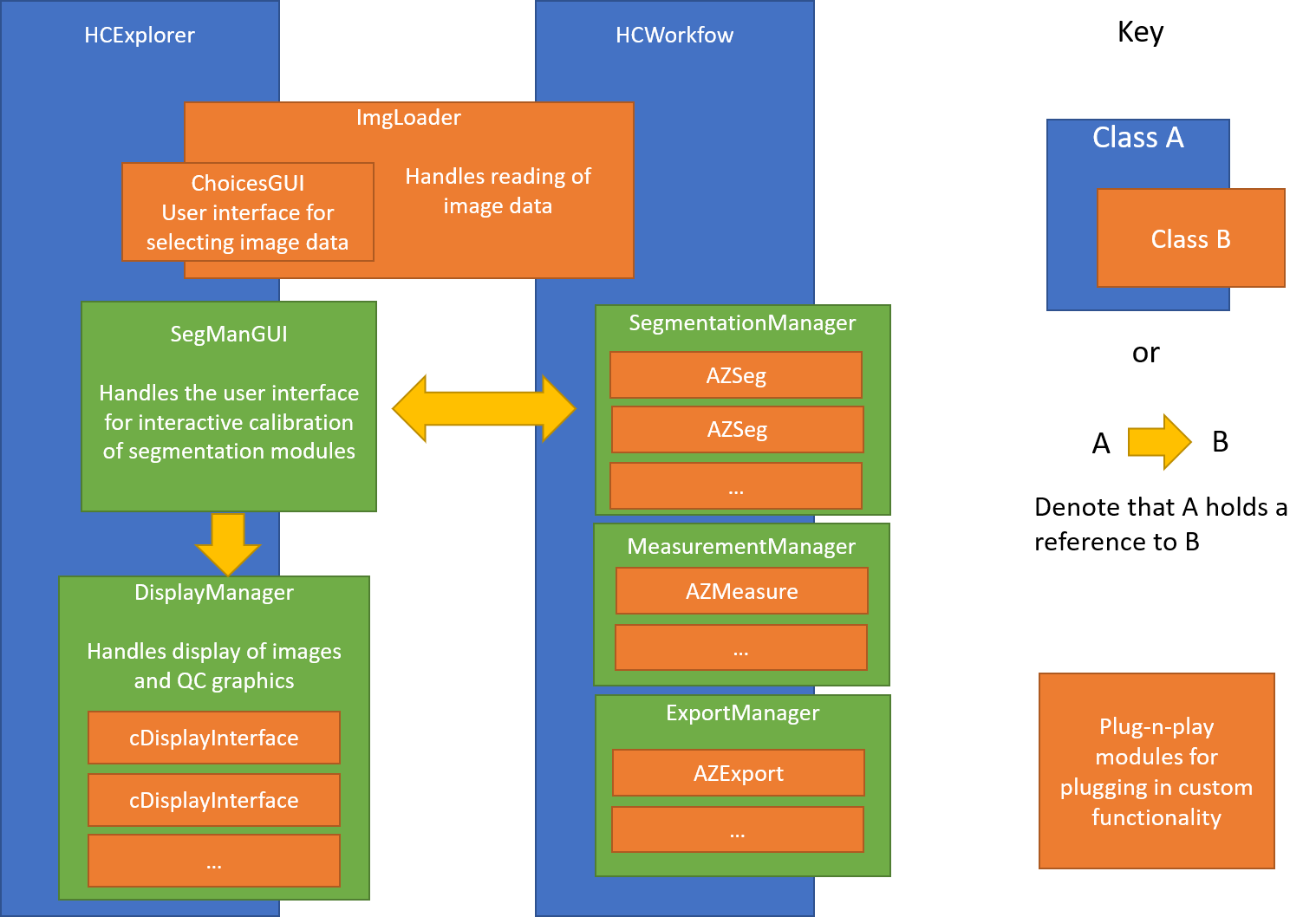


# Structure of a Workflow

function wk = nuclearPropertiesWorkflow(outputfolder,parserObj)

% Input parsing

if nargin<1

outputfolder = [];

end

if nargin<2

parserObj = [];

end

if ischar(parserObj)

parserObj = ParserYokogawa(parserObj);

end

nucchan = 1;

% Set up segmentation part of workflow

% Individual segmentation steps, eg nuclear segmentation, are written as

% classes. This allows common functionality, eg graphical interface for

% interactive parameter adjustment, to be stored in the parent class, and

% used by any segmentation classes, including newly written ones.

% A SegmentationManager handles which channels are passed to each

% segmentation stage, and organises the output label matrices

ss = SegmentationManager();

% A further reason for using a managing class to handle the input and

% output channels is so that the individual segmentation classes do not

% need to know about the overall experimental setup, and can therefore be

% used outside the framework on individual image and label matrices

% supply pre-processing options for each of the input channels

isettings = {'max','max','max'}; % make sure that the images are processed in 2D, even if they have z-slices

ss.supplyInputSettings(isettings);

% create a nuclear segmentation object with initial settings

% The input arguments are specific to each segmentation class - in this

% case the inputs are:

% 1) the typical size of nuclei

% 2) the intensity threshold

%

nuclearSeg = DoGNucAZSeg(16,0.08);

% the segmentation stage is then added to the segmentation manager, along

% with the image channel to be used. The third input, left empty here, is

% used if a prior label matrix is required for the segmentation (see

% cytoplasm segmentation below)

% SegmentationManager.addProcess(AZSeg object, input image channels, input label channels)

ss.addProcess(nuclearSeg,nucchan,[]);

% add a second segmentation object to segment the cell area

% In this experiment there is no cellular stain, so perform pseudo

% segmentation by expanding around the nuclear labels. Inputs are:

% 1) The distance by which to expand the labels

% 2) The weighting given to the image intensity, if an image channel is

% supplied

%

cytoSeg = PseudoCytoAZSeg(50,0.8);

% adding the cytoplasm segmentation to the manager.

% In this case, the image channel is left empty to denote that no image

% data is supplied, and the third input (1) denotes that the 1st label

% should be passed (ie the label output from the nuclear segmentation

% above)

% SegmentationManager.addProcess(AZSeg object, input image channels, input label channels)

ss.addProcess(cytoSeg,[],1);

% Set up the measurements

mm = MeasurementManager;

% Similar to the segmentation, measurements to be made from the images or

% segmentation results are handled by a MeasurementManager object, which

% handles the channels to be passed to each measurement class, and merges the

% outputs together into a single cell population structure

% create a measurement class to measure nuclear morphology statistics

% Typically for AZMeasure classes, the first input denotes a prefix to be

% added to the start of the names of measurements made by the class, to be

% stored in the output structure

nucMorphMeas = NucStatsAZMeasure('Nuc');

% For measurements, we supply the indices of the labels to be used (1 =

% nuclear segmentation result), and the image indices (DAPI channel)

% MeasurementManager.addMeasurement(AZMeasure object, input label channels, input image channels)

mm.addMeasurement(nucMorphMeas,1,nucchan);

% add further measurements

mm.addMeasurement(BasicIntensityAZMeasure('NucInt'),1,1:3);

mm.addMeasurement(BasicIntensityAZMeasure('Cyto'),2,1:3);

% Finally, an ExportManager handles the output of results as tables,

% mat-files and QC images

ee = ExportManager(outputfolder);

% the syntax for adding export objects is:

% ExportManager.addExporter(AZExport object, naming function, statsType)

% As a first export type, save the segmentation results in a mat-file

% MatLabelAZExport is a class which saves the label matrices to mat-files

% the second input argument is a handle to a naming function, which uses

% the information stored in each image object (Well location, field number,

% etc), to generate the filename for the save.

% As an example, multiLabelFile generates names in the format:

% /__labels/PLATE/label_A01_f1.mat

% This can be used as a template to create custom filename schemes.

ee.addExporter(MatLabelAZExport(),@multiLabelFile);

% Add export of the measurements to mat-files

% Inputs

% 1) AZExport object

% 2) naming function

% 3) which measurements to export

% The third input is used to determine which set of measurements should be

% saved, the options are 'SingleCell','Field', or 'Both'

ee.addExporter(MatStatsAZExport(),@multiStatsFile,'both');

% Export results to csv file (in this case tab-separated-value)

% Second argument specifies the naming function (here appending results to a single

% file)

ee.addExporter(DelimitedExport(),@multiCSVFile_OneFile);

% also include QC images for each segmentation step

% The QCImageAZExport class exports images overlaid with segmentation

% results, the syntax is:

% QCImageAZExport(image channel(s), segmentation label channel(s), image colours RGB, label colours RGB)

QC1 = QCImageAZExport(1,1,[1,1,1],[0.4,0.4,1]);

% the naming function determines the filename as well as the image type, in

% this case the ixQCFile generates png filenames

ee.addExporter(QC1,@(x)ixQCFile(x,'Nuc_'))

% export QC of cell segmentation

QC2 = QCImageAZExport([1,2],2,{[0,0,1];[0,1,0]},[1,0.4,1]);

ee.addExporter(QC2,@(x)ixQCFile(x,'Cell_'))

% at the end the managers are brought together into a workflow

% the HCWorkFlow object encapsulates the code for batch running, running in

% parallel, and linking together the steps of the workflow

wk = HCWorkFlow(ss,mm,ee,[],'Nuclear properties Assay');

% A parser object stores the architecture of the imaging experiment. If we

% know what experiment the workflow will be applied to, it can be added to

% the workflow here

if ~isempty(parserObj)

wk.addParser(parserObj);

end

# Anatomy of a Module

% Basic nuclei detection, based on smoothing and thresholding

%> @file BasicNucAZSeg.m

%> @brief Basic nuclei detection, based on smoothing and thresholding

%> @brief Tuneable parameters are the approximate radius of nuclei, and relative threshold

%> The nuclear radius is used to split touching objects using a distance transform

%> The relative threshold is the adjustment relative to threshold calculated using Otsu's method

classdef BasicNucAZSeg < AZSeg

% Basic nuclei detection

% Based upon smoothing, thresholding and then breaking apart touching nuclei

Adjustable parameters are stored as class properties, but properties must also be highlighted below to be flagged as adjustable

properties

%> Radius of nuclei

NucRadius = 32 % rough radius of desired objects

%> Threshold adjustment relative to Otsu level

RelThresh = 0 % threshold relative to Otsu

end

methods

% ======================================================================

%> @brief Class constructor

%>

%> Return the segmentation object with initial values for tuneable parameters

%>

%> @param nucrad Radius of nuclei in pixels

%> @param pthr Threshold adjustment relative to Otsu level

%>

%> @return instance of the BasicNucAZSeg class.

% ======================================================================

function this = BasicNucAZSeg(nucrad,pthr)

Specify which class properties are adjustable parameters here, and how we want them to appear in the calibration GUI

% set up the interactive parameter tuning

this = this@AZSeg({'NucRadius','RelThresh'},...

{'Radius of Nuclei','Threshold Adjustment'},...

'Nucleus Detection',...

1,... Number of input channels

0,... Number of input segmentation masks

1); % Number of output segmentation masks

% 1,0,1 is the default, could be left out

if nargin>0 && ~isempty(nucrad)

this.NucRadius = nucrad;

end

if nargin>1 && ~isempty(pthr)

this.RelThresh = pthr;

end

end

% ======================================================================

The process method is called for running the analysis. This is where the custom image processing code is placed

%> @brief Run the nuclear segmentation

%>

%>

%> @param this instance of the BasicNucAZSeg class

%> @param im image data (single channel, 2D)

%> @param ~ no label data required

%>

%> @return L label matrix of detected nuclei

% ======================================================================

function L = process(this,im,~,~)

% this kind of input checking can be farmed out to specialized

% superclasses

if iscell(im)

im = im{1};

end

if ~isa(im,'double')

im = double(im);

end

% link the smoothing scale to the nuclear size

smoothScale = max(1,this.NucRadius/4);

blim = amcBilateral2D(sqrt(im),[],smoothScale,0.1,16,4);

% For fixed images, a basic threshold can be enough

bw = blim>((1+this.RelThresh)*amcGrayThresh(blim));

% morphological smoothing

bwoc = imclose(imopen(bw,diskElement(8)),diskElement(8));

bwoc2 = openCloseByRecon(bw,diskElement(8));

bwuse = bwoc & bwoc2;

D = bwdist(~bwuse);

smD = gaussFiltND(D,0.5*this.NucRadius*[1,1]);

dogD = smD - gaussFiltND(D,0.5*this.NucRadius*[1.6,1.6]);

dogD = imreconstruct(dogD-0.5,dogD);

lmax = imregionalmax(dogD) & D>5;

% newbw = imreconstruct(lmax,bwuse);

Dbg = bwdist(bwuse);

% bg = bwmorph(~bwuse,'skel',Inf);

bg = bwmorph(~bwuse,'skel',10);

L = {double(markerWatershed(-D-Dbg,lmax,bg))};

end

end

methods (Static)

% ======================================================================

%> @brief get a description of the image processing operation to be displayed in GUI components

%>

%> @return str cell array of strings containing description of operation

% ======================================================================

function str = getDescription()

str = {'Segmentation of fixed nuclei','',...

['Detect nuclei that have been stained by DAPI, Hoechst or similar.',...

' Basic smoothing and thresholding, followed by attempting to break',...

' apart touching nuclei.']};

end

A description to appear in the calibration GUI is specified by the getDescription static method.

end

end
