# Supplementary material for: IA-Lab: A MATLAB framework for efficient microscopy image analysis development, applied to quantifying intracellular transport of internalized peptide-drug conjugate
Source: PLoS One. 2019 Aug 1;14(8):e0220627. doi: 10.1371/journal.pone.0220627 (PMC6675096; doi:10.1371/journal.pone.0220627)
Supplement: S3 File — List of currently available workflow modules with brief description. (DOCX) [file pone.0220627.s003.docx]

Available workflow modules

# Segmentation Modules

AZSeg - interface for segmentation classes

BasicNucAZSeg - Basic nuclei detection, based on smoothing and thresholding

CellMaskAZSeg - expand around a foreground marker to find the cell cytoplasm in 2D

ClusterGradMaskAZSeg - same procedure as the cluster nuclei, but starting from the nuclear

CytoFibreAZSeg - expand around nuclei using the inverse of intensity as a distance

CytoMonolayer3DAZSeg - Perform 3D segmentation of 3D cells in a monolayer from cellmask and nuclear labels

CytoRoughAZSeg - expand around nuclei using the inverse of intensity as a distance

DenseCellMaskAZSeg - expand around nuclei using the inverse of intensity as a distance

DenseCellNoNucAZSeg - Densely clustered cells with cell marker but no nucleus marker.

DenseNucAZSeg - Difference of Gaussians to find centres, gradient watershed for segmentation.

DoGNucAZSeg - Segmentation of nuclei based on gradient of intensity at edges

FaintNucAZSeg - first build of segmentation class, taking faint nuclei as an example

GradNucAZSeg - Nuclear segmentation - finding regional maxima and separating them using gradient watershed.

LabelCombineAZSeg - Combine two labels, using the specified function

LabelMorphAZSeg - Expansion or contraction of label regions from the boundary

LowMagNucAZSeg - Segmentation of small and clustered nuclei

MicroNucleiAZSeg - Detect micronuclei, separate from the main nucleus

NucMonolayer3DAZSeg - Perform 3D segmentation of nuclei in a monolayer from a 3D image

OneStageAZSeg - sub class for a single stage of segmentation, with a possible

PseudoCytoAZSeg - Pseudo-cytoplasm segmentation

SegmentationManager - first attempt at stand-alone segmentation manager

SpotDetect3DAZSeg - Detection of spots (eg DNA damage, FISH transcription spots) in 3D, within cells/objects.

SpotDetect3DNoLabelAZSeg - Detection of spots (eg DNA damage, FISH transcription spots) in 3D, without masking by cell/object region.

TestNucAZSeg - first build of segmentation class, taking faint nuclei as an example

ThresholdAZSeg - Basic thresholding and labelling - don't expect this will be very useful.

TwoStageAZSeg - Sub class for segmentation cases which can be logically separated into two

TwoStageSeedAZSeg - two stages of segmentation, for instance nuclei segmentation followed

# Measurement Classes

AZMeasure - Interface for measurement classes

AZMeasurePixels - parent class allowing pixel size information to be passed to the measurement class.

BasicIntensityAZMeasure - Measure the mean intensity, max and min, and also area so that total intensity can be calculated.

BasicMeasure_Cell - Basic shape measurements - area, aspect, solidity, etc

BlurMetricAZMeasure - operate on a whole image, to determine if there is a lot of blurring

CentroidIntensityAZMeasure - take the intensity at the centroid of the segmented regions

DelaunayEntropyAZMeasure - Measure cell organisation using the entropy of Delaunay triangle areas.

IntensityFocusAZMeasure - Measure how focussed the intensity

Measure_OpticArtefacts - Calculate the magnitude of optical artefacts in the image

MeasureExtractCellomics - Extract Cellomics segmentation results from the object files

MeasurementManager - Manager for the measurements, which passing the correct channels to each measurement module and combining the outputs.

MicroNucleiAZMeasure - Calculate statistics of micro-nuclei - area, intensity and distance from the nucleus.

NucStats3DAZMeasure - Measure Nuclear properties from 3D labels

NucStatsAZMeasure - Measure Nuclear properties, including total DNA content for cell-cycle.

PixelClusterAZMeasure - (Deprecated) Measure the extent of clustering from the radial distribution function.

RingIntensityAZMeasure - Measure intensity in rings inside and outside the nucleus (or other objects).

ShapeBinsAZMeasure - Statistics reflecting the cell shape, using the histogram of the cell boundary distance transform.

ShapeStatsAZMeasure - Measure shape properties for the segmented regions

Spheroid2DStatsAZMeasure - Measure shape properties for the segmented spheroid (note that this expects one spheroid per image).

SpotCountAZMeasure - count how many spots (label 2) are in each object (label 1)

SpotStatsAZMeasure - Statistics of spots (label 2) in each object (label 1) (number, location, intensity).

SubcellIntensityAZMeasure - Intensity by subdividing the cell into regions based on the distance from the cell edge.

SubNucCellIntensityAZMeasure - Intensity by subdividing the cell into regions based on the distance from the cell and nucleus edges.

TouchingEdgeAZMeasure - How much of each label is touching the border of the image.
