# Supplementary material for: IA-Lab: A MATLAB framework for efficient microscopy image analysis development, applied to quantifying intracellular transport of internalized peptide-drug conjugate
Source: PLoS One. 2019 Aug 1;14(8):e0220627. doi: 10.1371/journal.pone.0220627 (PMC6675096; doi:10.1371/journal.pone.0220627)

Additional analysis workflows

## 3D

Analysis can be done on 3D image stacks, working in true 3D and not just one 2D image slice at a time. In 3D, as in 2D, images can be browsed and analyzed using the plate map, or batch analysis for the whole plate can be run.


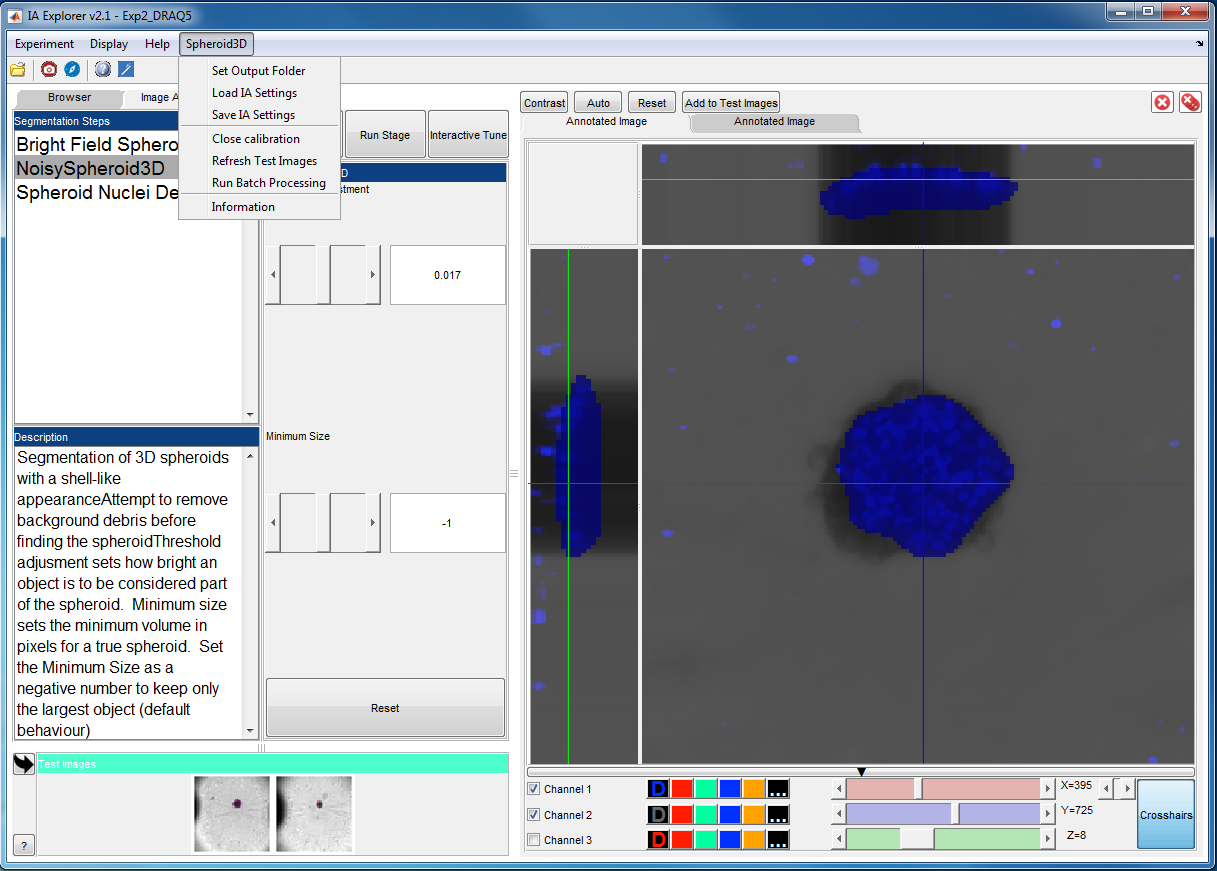

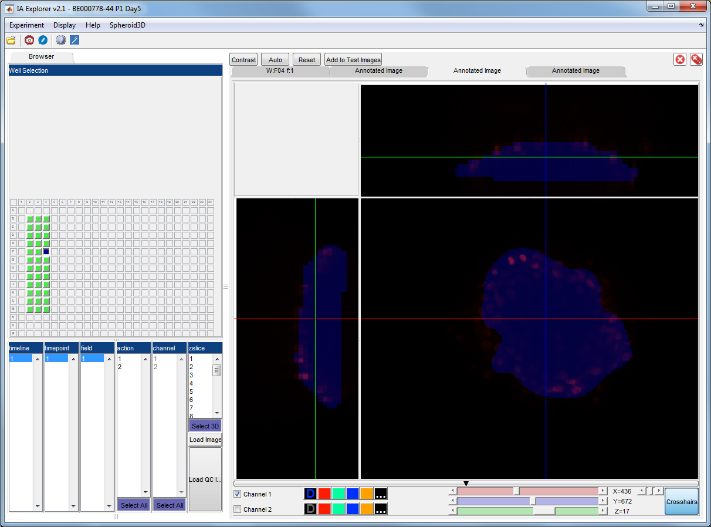


## Non-microscopy images

Although developed primarily for plate based microscopy image analysis, the tool is very general, and any image sets can be analyzed, including images from regular camera photography. Workflows can be fully automatic for whole image sets, or can include manual fine tuning when needed.


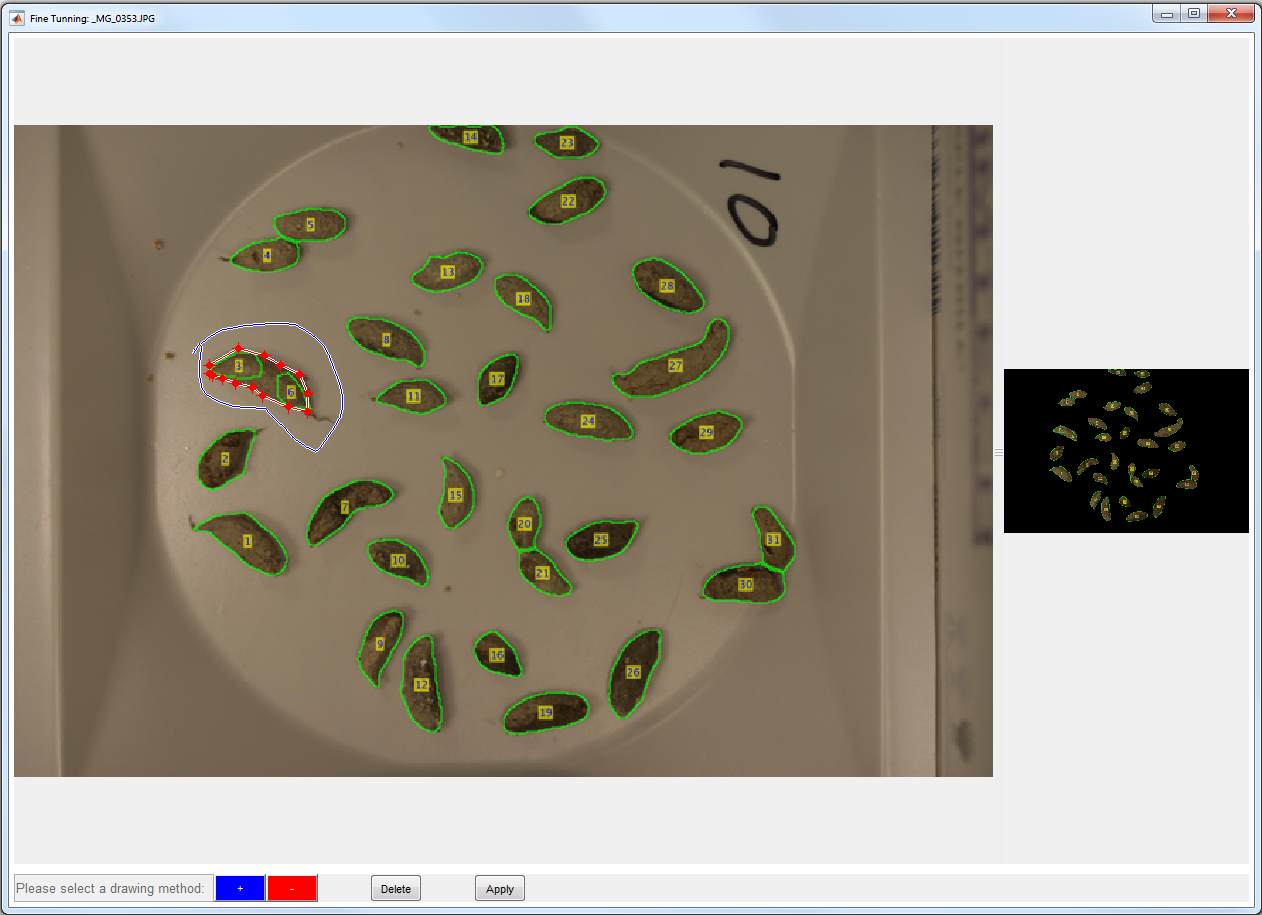


To run this example, please follow these steps:

To view images:

1. Type in pelletPoC in Matlab command window to launch the user interface.
2. Click
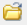
 to navigate to the location where the image folder is.
3. Highlight an image in the left hand side image list, e.g.
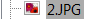
, then click the
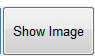
 button to show an image.

To analyze images:

1. In the menu bar, click “Image Analysis -> Setup Result Directory” first, to create a folder where you want to save analysis results.
2. Click “Image Analysis -> Run Current Image” or, “Ctrl + R” to perform image analysis on the current image.
3. When results are show on the right hand side viewer, you can click the
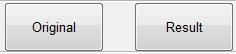
 buttons to view either the original or the marked up images.
4. If you are unhappy with the results, then click “Image Analysis -> Fine Tune” or, “Ctrl + T” to load up the Fine Tune window, where you can correct the segmentation of individual object.
5. If there are objects not been recognized, you can then click the
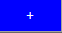
 button to add a new annotation. If background were wrongly recognised as a foreground object, then click the
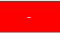
 button to remove it. The right hand side viewer in this Fine Tune window will the edited effect. You can also drag the
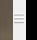
 bar in the middle of the two images to adjust image sizes.
6. Click the
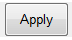
 button to apply the changes.
7. Numerical results are stored in the result directory specified in step 1. [FileName]_Results.csv stores the image level results. [FileName]_Individual Results.csv stores the measurements for each segmented object, where each row in the csv file corresponds to a single segmented object.

# Nuclear Properties Workflow

## Walkthrough for standard screen

## Code Structure

The code in /Examples/nucleiExample.m details how to launch the workflow within the GUI:

function ia = nucleiExample()

% the function nuclearPropertiesWorkflow.m returns a HCWorkFlow object.

% the function creates each of the analysis steps and adds them to the

% workflow.

wk = nuclearPropertiesWorkflow();

% The GUI is then invoked, and the workflow attached to the GUI object

ia = HCExplorer().addWorkflow(wk);

% optionally quit MATLAB upon exit - uncomment the code below

% % waitfor(ia.Figure1)

% % quit()

end

The function nuclearPropertiesWorkflow.m creates the workflow by adding segmentation, measurement and export modules, defining which channels are used for each, and setting up the starting values for the tuneable parameters:

function wk = nuclearPropertiesWorkflow(outputfolder,parserObj)

% Input parsing

if nargin<1

outputfolder = [];

end

if nargin<2

parserObj = [];

end

if ischar(parserObj)

parserObj = ParserYokogawa(parserObj);

end

nucchan = 1;

% Set up segmentation part of workflow

% Individual segmentation steps, eg nuclear segmentation, are written as

% classes. This allows common functionality, eg graphical interface for

% interactive parameter adjustment, to be stored in the parent class, and

% used by any segmentation classes, including newly written ones.

% A SegmentationManager handles which channels are passed to each

% segmentation stage, and organises the output label matrices

ss = SegmentationManager();

% A further reason for using a managing class to handle the input and

% output channels is so that the individual segmentation classes do not

% need to know about the overall experimental setup, and can therefore be

% used outside the framework on individual image and label matrices

% supply pre-processing options for each of the input channels

isettings = {'max','max','max'}; % make sure that the images are processed in 2D, even if they have z-slices

ss.supplyInputSettings(isettings);

% create a nuclear segmentation object with initial settings

% The input arguments are specific to each segmentation class - in this

% case the inputs are:

% 1) the typical size of nuclei

% 2) the intensity threshold

%

nuclearSeg = DoGNucAZSeg(16,0.08);

% the segmentation stage is then added to the segmentation manager, along

% with the image channel to be used. The third input, left empty here, is

% used if a prior label matrix is required for the segmentation (see

% cytoplasm segmentation below)

% SegmentationManager.addProcess(AZSeg object, input image channels, input label channels)

ss.addProcess(nuclearSeg,nucchan,[]);

% add a second segmentation object to segment the cell area

% In this experiment there is no cellular stain, so perform pseudo

% segmentation by expanding around the nuclear labels. Inputs are:

% 1) The distance by which to expand the labels

% 2) The weighting given to the image intensity, if an image channel is

% supplied

%

cytoSeg = PseudoCytoAZSeg(50,0.8);

% adding the cytoplasm segmentation to the manager.

% In this case, the image channel is left empty to denote that no image

% data is supplied, and the third input (1) denotes that the 1st label

% should be passed (ie the label output from the nuclear segmentation

% above)

% SegmentationManager.addProcess(AZSeg object, input image channels, input label channels)

ss.addProcess(cytoSeg,[],1);

% Set up the measurements

mm = MeasurementManager;

% Similar to the segmentation, measurements to be made from the images or

% segmentation results are handled by a MeasurementManager object, which

% handles the channels to be passed to each measurement class, and merges the

% outputs together into a single cell population structure

% create a measurement class to measure nuclear morphology statistics

% Typically for AZMeasure classes, the first input denotes a prefix to be

% added to the start of the names of measurements made by the class, to be

% stored in the output structure

nucMorphMeas = NucStatsAZMeasure('Nuc');

% For measurements, we supply the indices of the labels to be used (1 =

% nuclear segmentation result), and the image indices (DAPI channel)

% MeasurementManager.addMeasurement(AZMeasure object, input label channels, input image channels)

mm.addMeasurement(nucMorphMeas,1,nucchan);

% add further measurements

mm.addMeasurement(BasicIntensityAZMeasure('NucInt'),1,1:3);

mm.addMeasurement(BasicIntensityAZMeasure('Cyto'),2,1:3);

% Finally, an ExportManager handles the output of results as tables,

% mat-files and QC images

ee = ExportManager(outputfolder);

% the syntax for adding export objects is:

% ExportManager.addExporter(AZExport object, naming function, statsType)

% As a first export type, save the segmentation results in a mat-file

% MatLabelAZExport is a class which saves the label matrices to mat-files

% the second input argument is a handle to a naming function, which uses

% the information stored in each image object (Well location, field number,

% etc), to generate the filename for the save.

% As an example, multiLabelFile generates names in the format:

% /__labels/PLATE/label_A01_f1.mat

% This can be used as a template to create custom filename schemes.

ee.addExporter(MatLabelAZExport(),@multiLabelFile);

% Add export of the measurements to mat-files

% Inputs

% 1) AZExport object

% 2) naming function

% 3) which measurements to export

% The third input is used to determine which set of measurements should be

% saved, the options are 'SingleCell','Field', or 'Both'

ee.addExporter(MatStatsAZExport(),@multiStatsFile,'both');

% Export results to csv file (in this case tab-separated-value)

ee.addExporter(DelimitedExport(),@multiCSVFile_OneFile);

% also include QC images for each segmentation step

% The QCImageAZExport class exports images overlaid with segmentation

% results, the syntax is:

% QCImageAZExport(image channel(s), segmentation label channel(s), image colours RGB, label colours RGB)

QC1 = QCImageAZExport(1,1,[1,1,1],[0.4,0.4,1]);

% the naming function determines the filename as well as the image type, in

% this case the ixQCFile generates png filenames

ee.addExporter(QC1,@(x)ixQCFile(x,'Nuc_'))

% export QC of cell segmentation

QC2 = QCImageAZExport([1,2],2,{[0,0,1];[0,1,0]},[1,0.4,1]);

ee.addExporter(QC2,@(x)ixQCFile(x,'Cell_'))

% at the end the managers are brought together into a workflow

% the HCWorkFlow object encapsulates the code for batch running, running in

% parallel, and linking together the steps of the workflow

wk = HCWorkFlow(ss,mm,ee,[],'Nuclear properties Assay');

% A parser object stores the architecture of the imaging experiment. If we

% know what experiment the workflow will be applied to, it can be added to

% the workflow here

if ~isempty(parserObj)

wk.addParser(parserObj);

end

A workflow set up in this way can make use of the framework tools for image browsing, parameter tuning, QC of analysis and batch running (see next section).

Alternatively, the batch run can be carried out from the command line, using the methods runBatch to use a single core or parRunBatch for parallelised batch analysis.

## Using the GUI

1. Ensure that the repository and subfolders are added to the MATLAB path, and run the following in the command window:

ia = nucleiExample();

1. Select Experiment>New Experiment>Yokogawa.. from the menu of the window that appears:
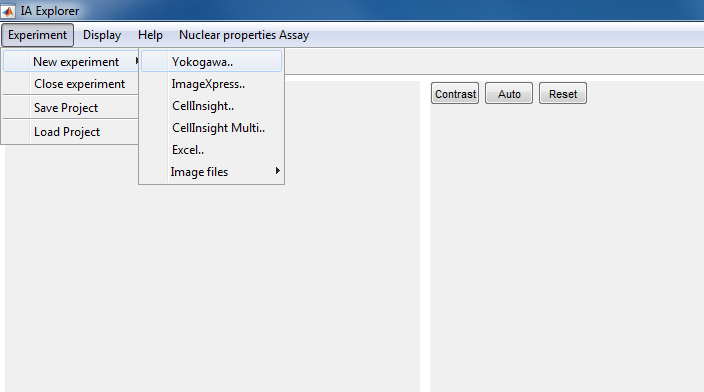

2. Navigate to the folder that contains the experiment images (for this example, they are stored in Examples\Nuclei\AssayPlate within the repository:
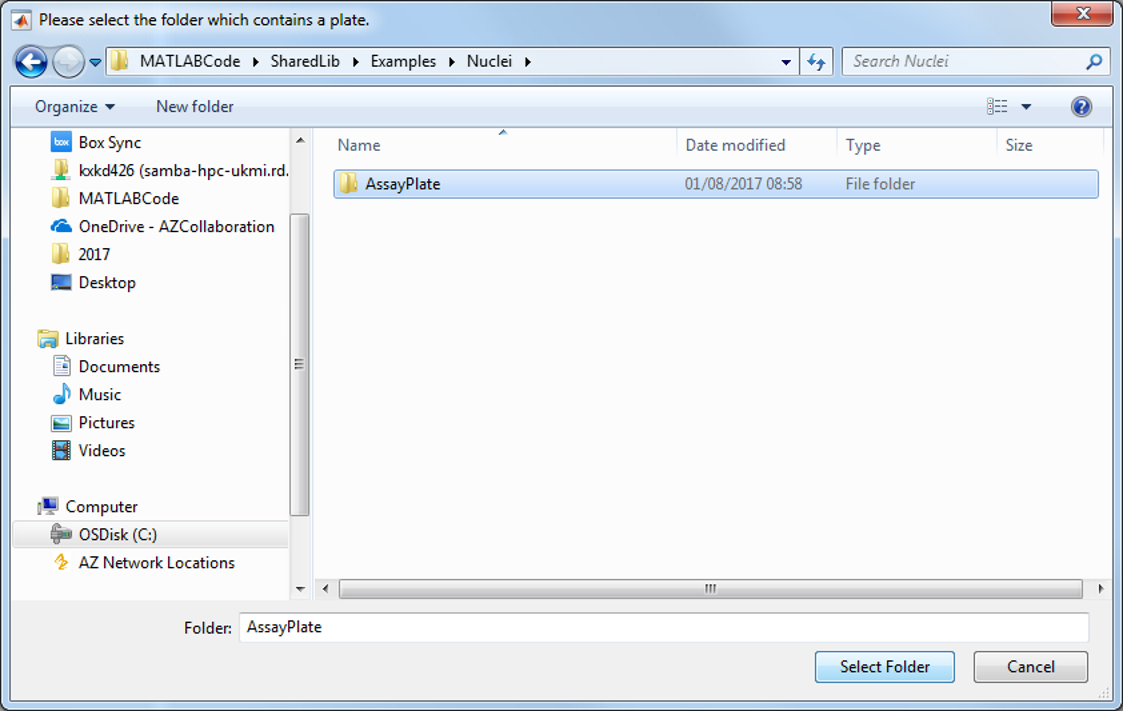

3. The plate map and image hierarchy for the experiment appears in the ‘browser’ panel on the left:
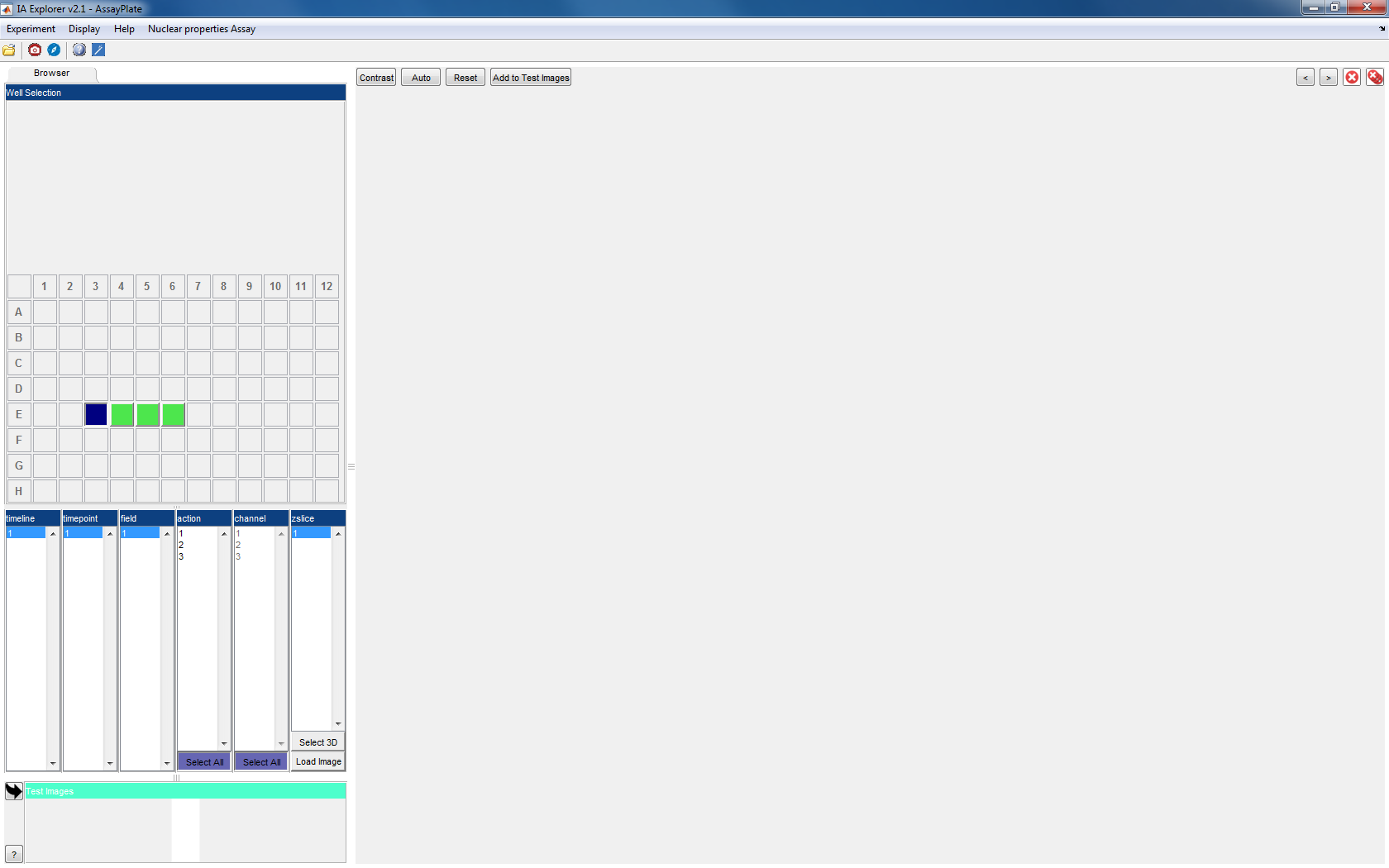

4. Select an appropriate well (or keep the initial selection) and click ‘load image’ at the bottom of the browser panel. The image is displayed on the right hand side:
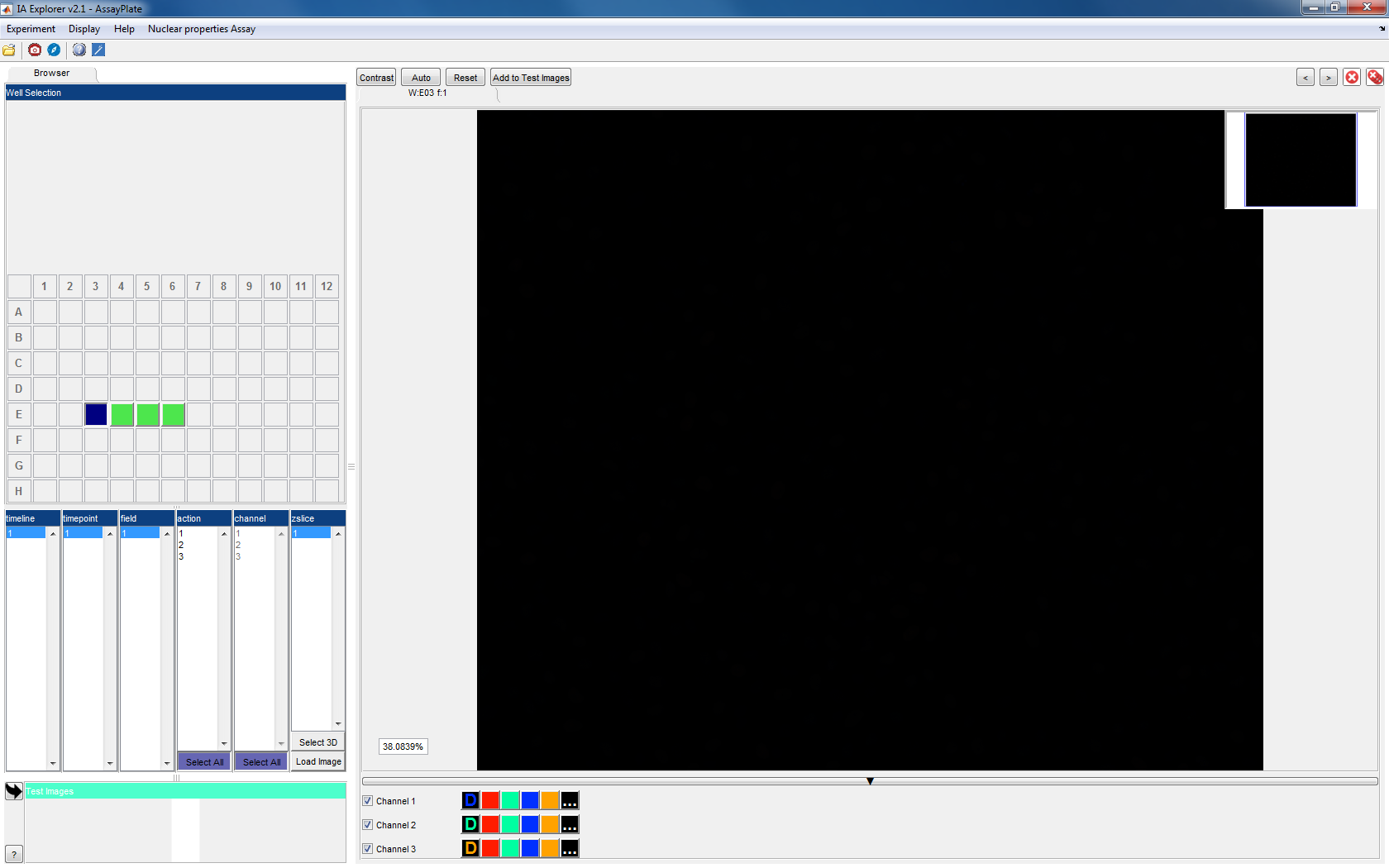

5. The image is shown without any contrast adjustment – click the ‘Auto’ button at the top of the display to automatically adjust the contrast (the ‘Contrast’ button allows manual fine tuning of the display contrast)
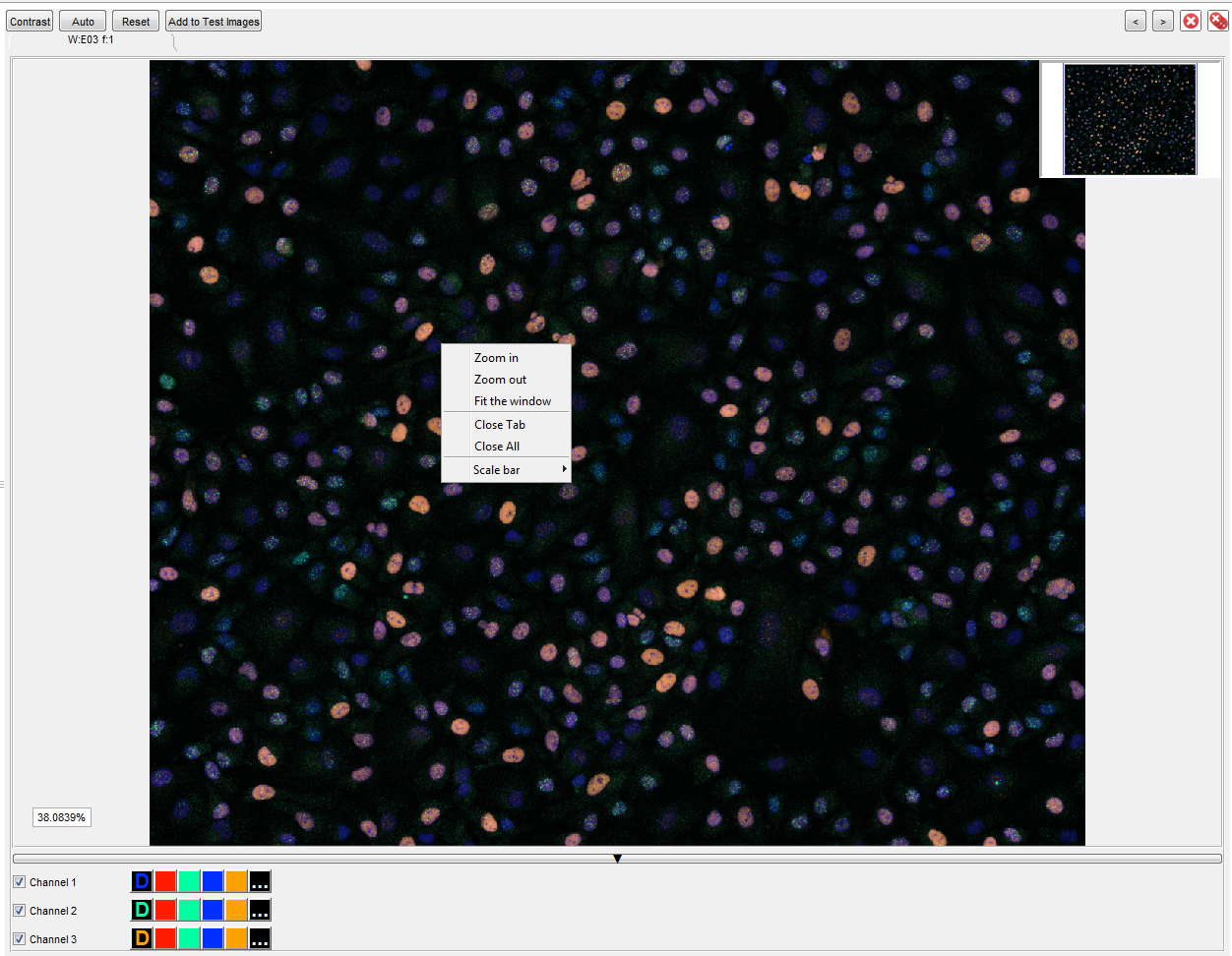
To adjust the image magnification, right click and choose zoom in or zoom out, or use the mouse scroll wheel.
6. Set the output folder, where results of the batch run will be stored:
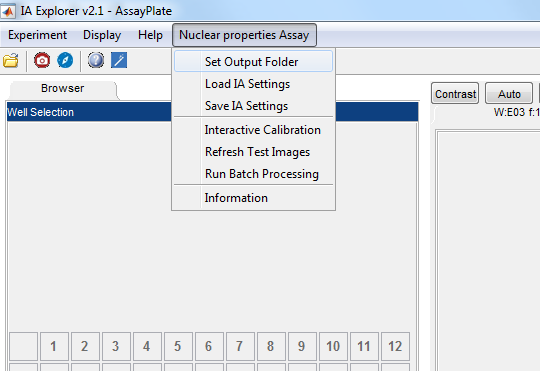

7. To adjust image processing settings, select Interactive Calibration from the Nuclear Properties Assay menu. This will run the image processing workflow on the currently selected image – to run the calibration on multiple images, use the ‘Add to Test Images’ button to build a set of images, before selecting interactive calibration
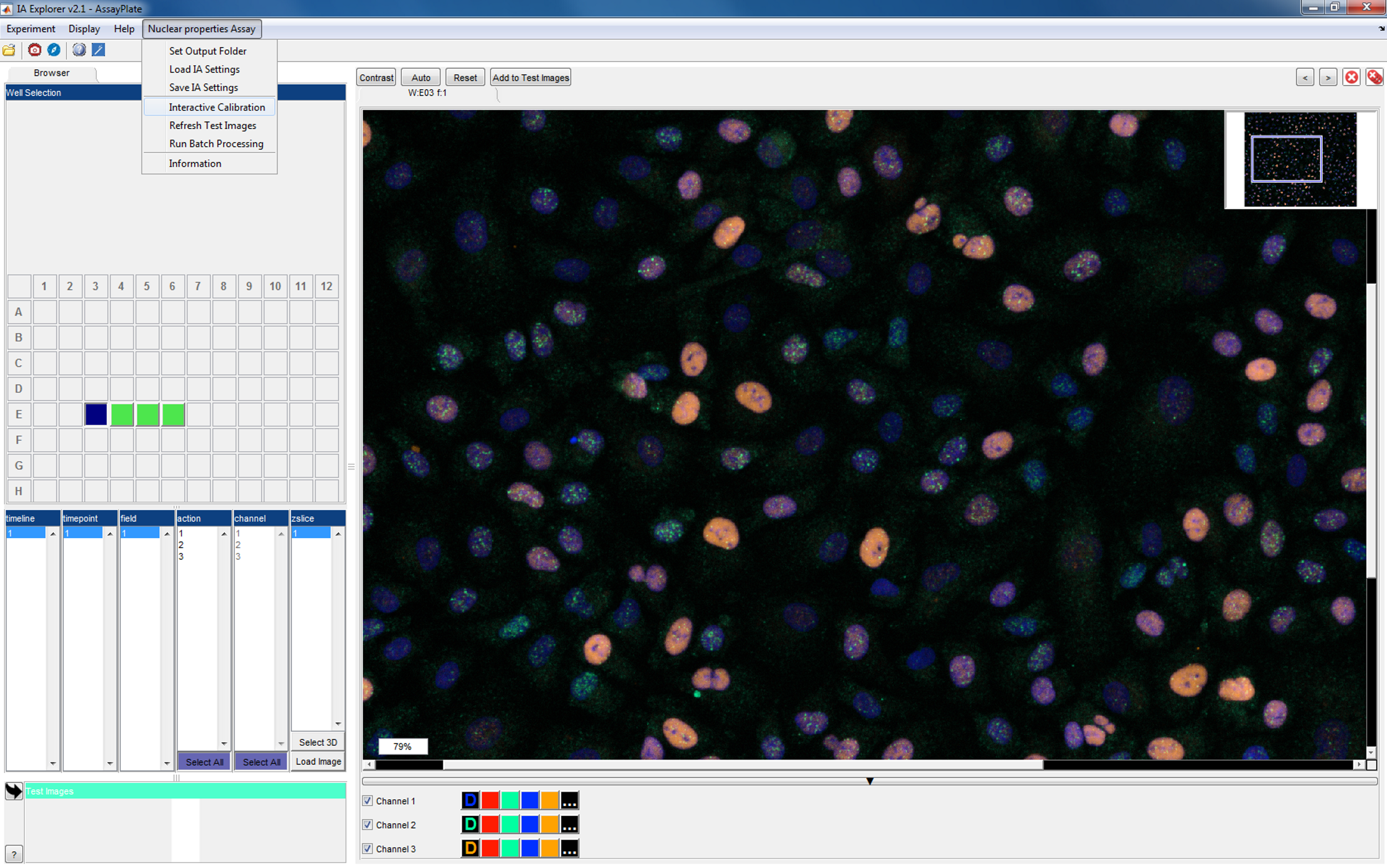

8. The window that appears lists the parameters than can be adjusted for each step of the image processing. The values can be adjusted using the sliders or the edit boxes. To apply changes to the settings, click the Apply stage button. To apply the changes and see the resulting segmentation, click the ‘Run Stage’ button:


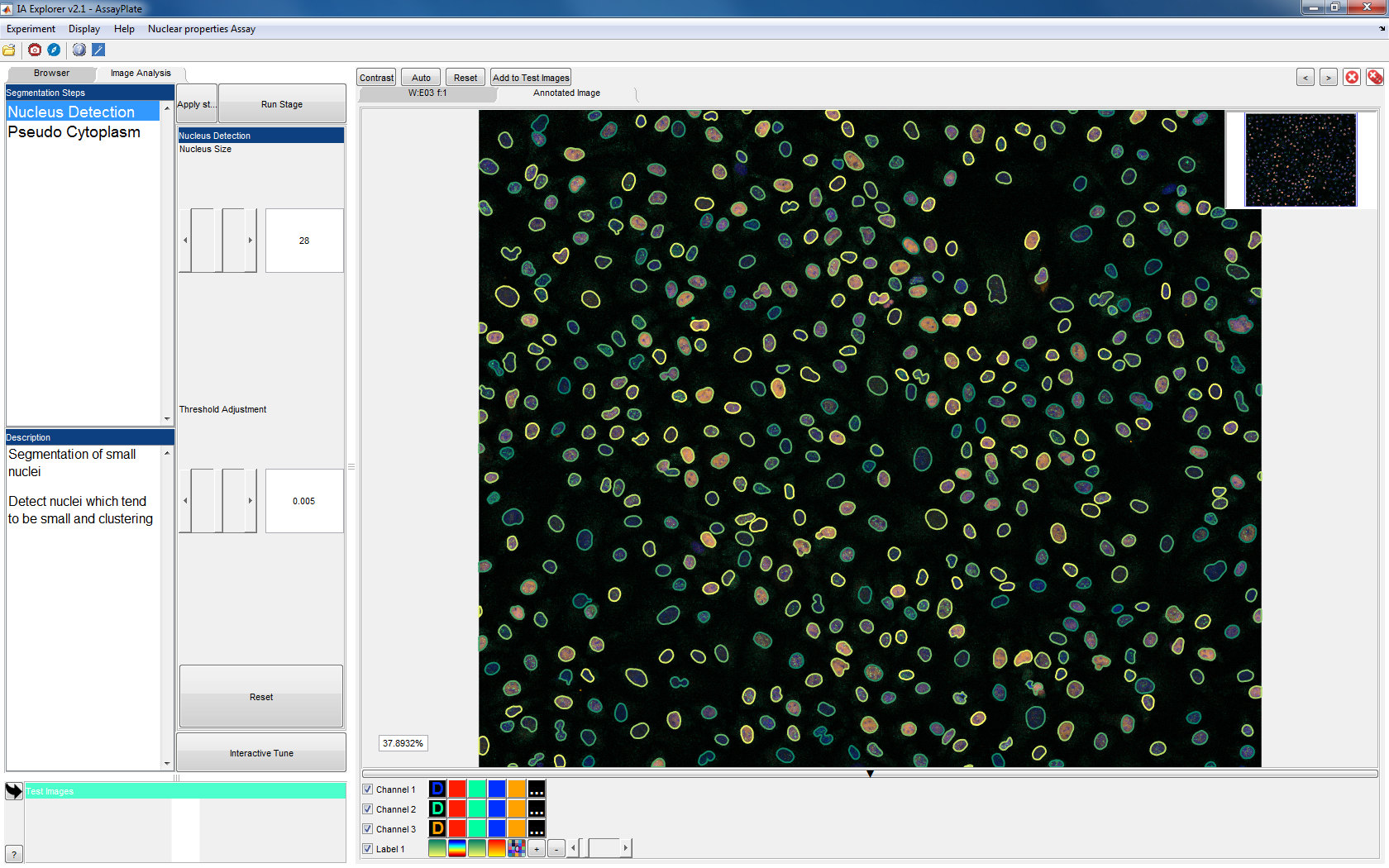


1. Select the second segmentation stage ‘Pseudo Cytoplasm’ in the left panel, and ‘Run Stage’:
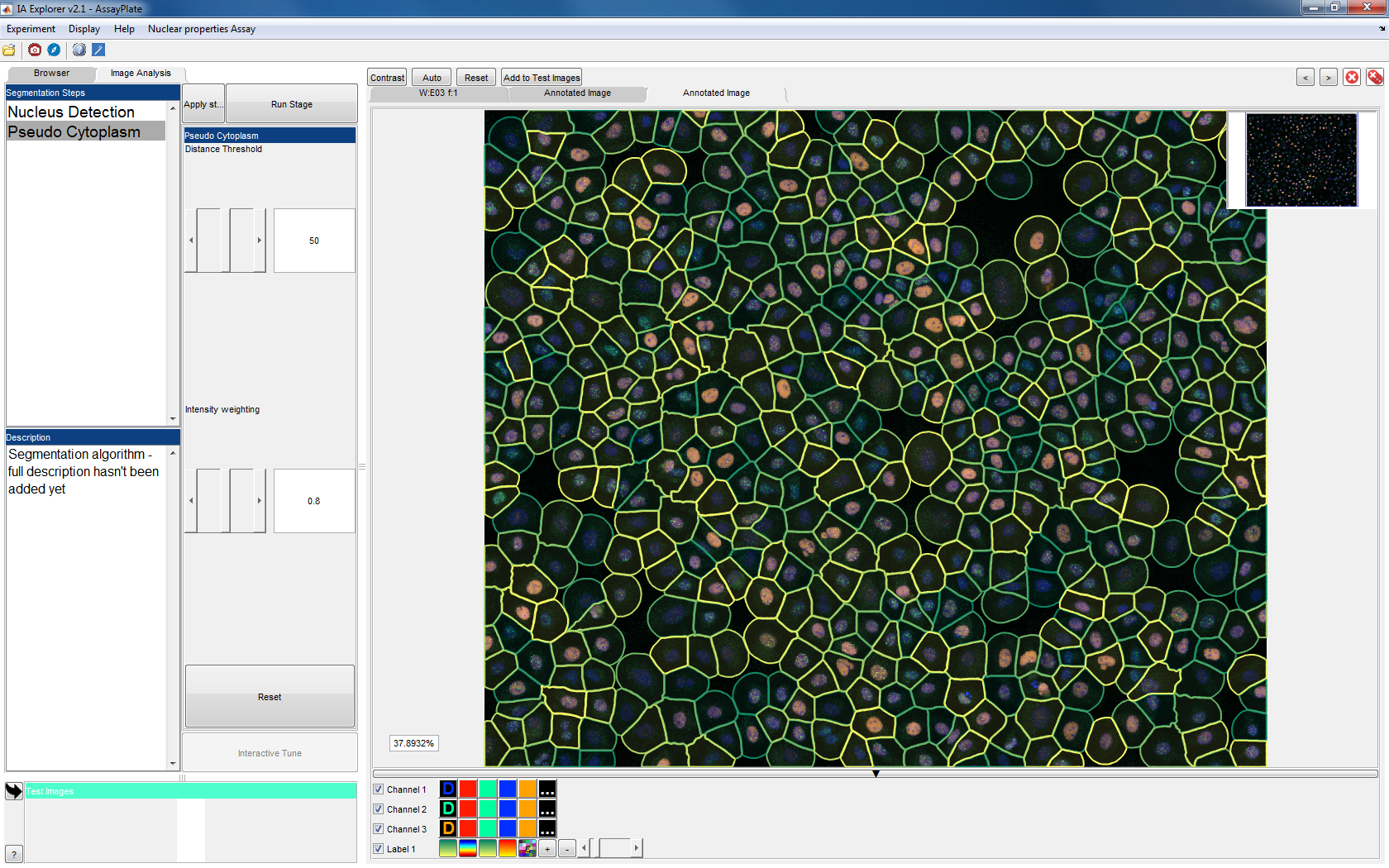

2. When happy with the settings, select ‘Run Batch Processing’ from the Assay menu, and follow the prompts:
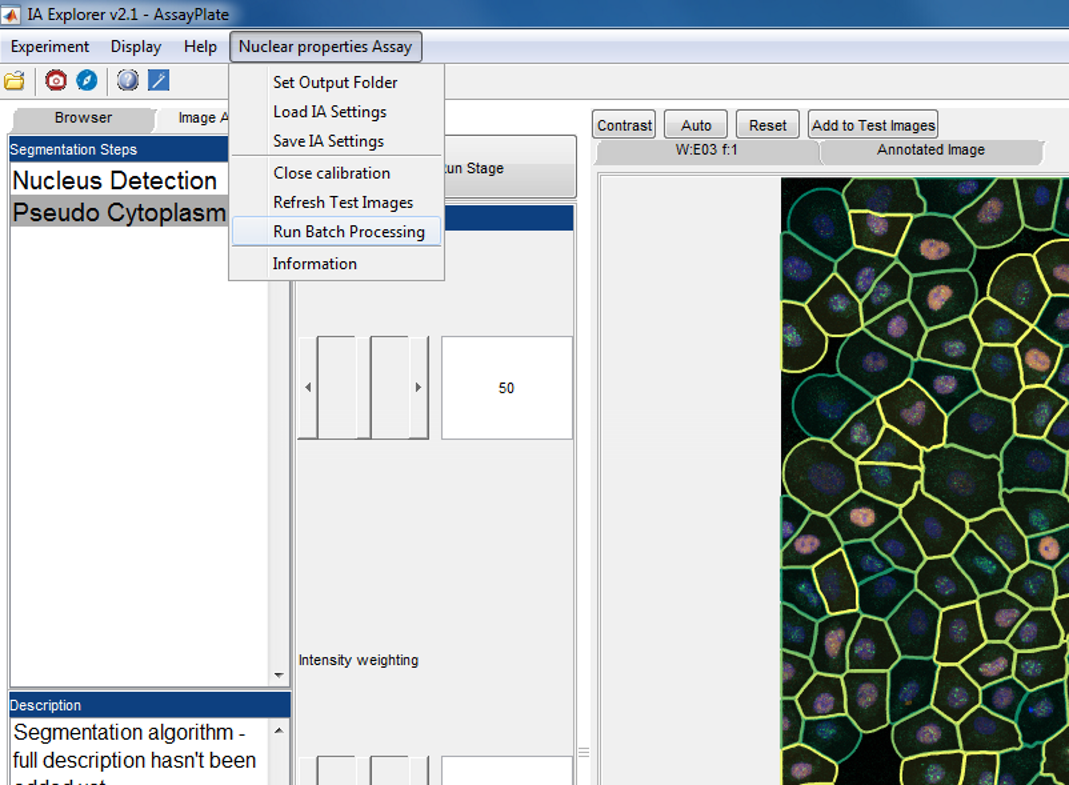


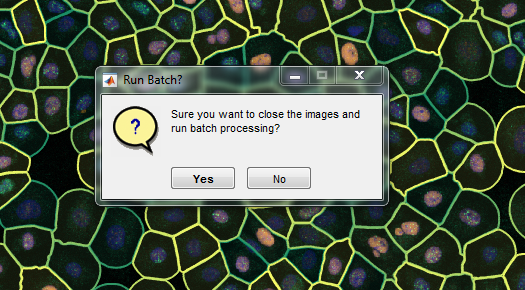


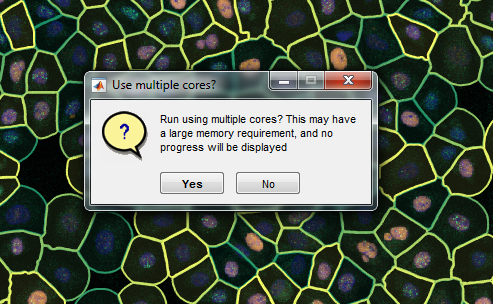


1. If run without parallelising, progress is displayed:
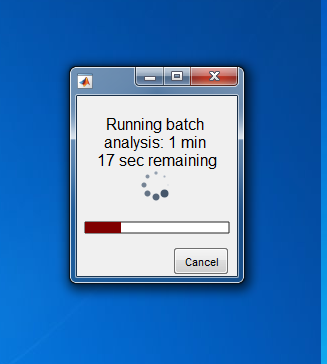

2. Results are stored in the chosen output folder:
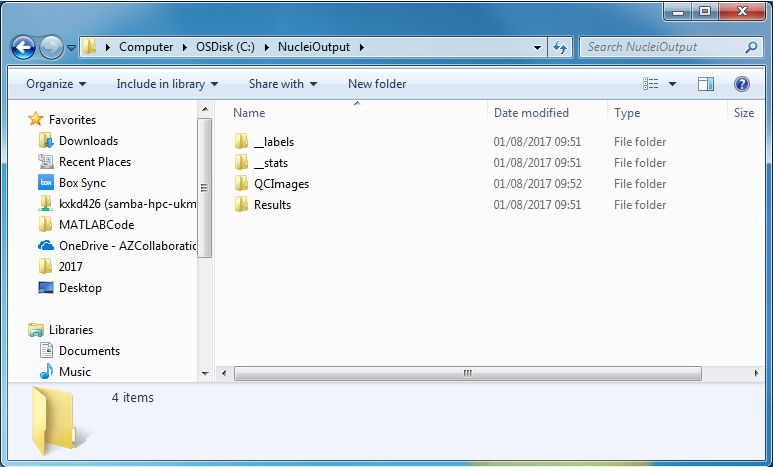
The folders __labels and __stats contain single cell results stored as .mat files. QCImages contains images showing the segmentation results. The measured single cell properties are stored as semicolon separated data file in the Results folder. This can be imported directly into one’s chosen analysis software.

This command reads the results into MATLAB (after setting current directory to the output folder) as a table:

T = readtable('Results/Results_AssayPlate.csv','delimiter',';');
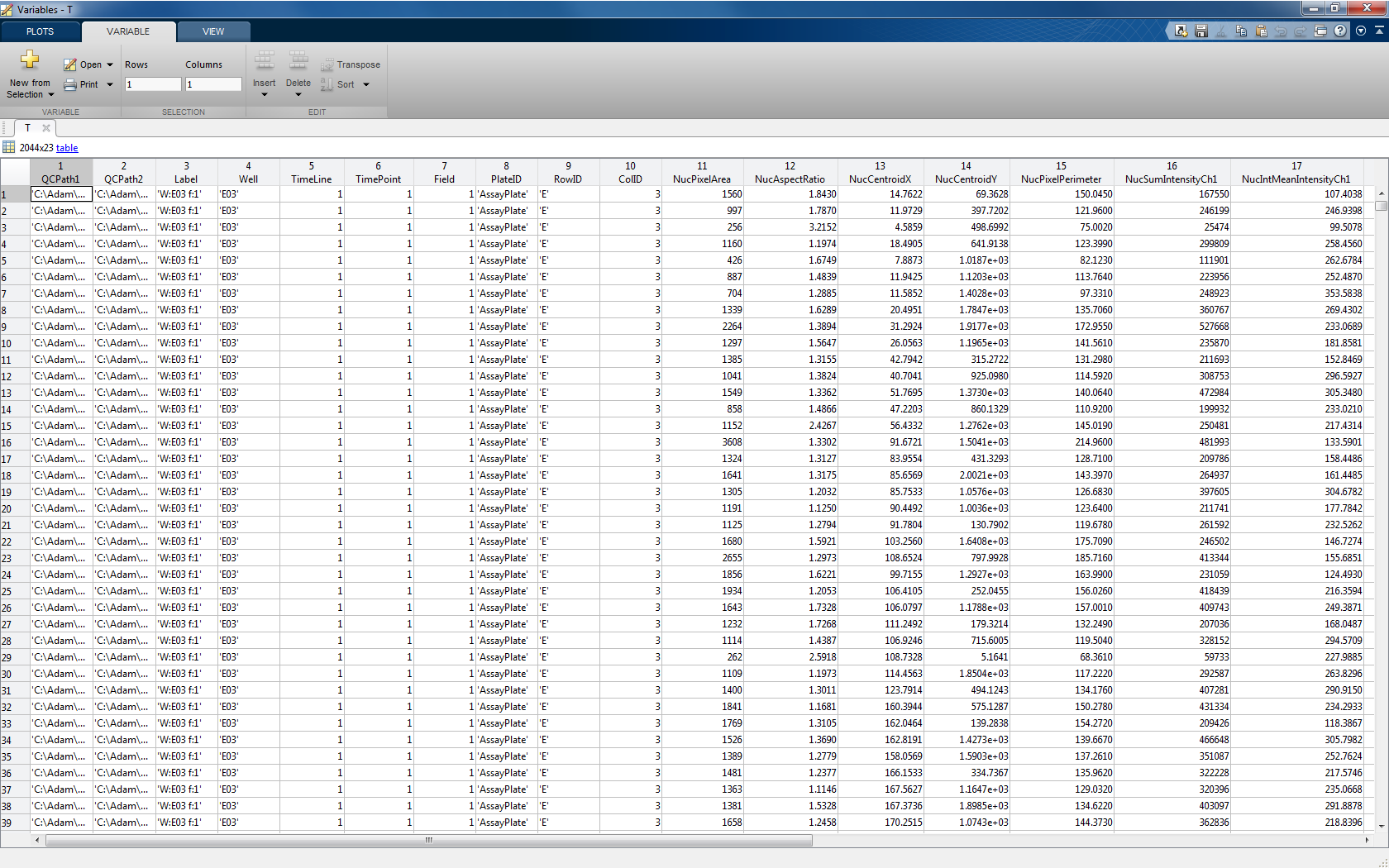

Supplement: S4 File — Further examples of different types of workflows. (DOCX) [file pone.0220627.s004.docx]
