# Supplementary material for: IA-Lab: A MATLAB framework for efficient microscopy image analysis development, applied to quantifying intracellular transport of internalized peptide-drug conjugate
Source: PLoS One. 2019 Aug 1;14(8):e0220627. doi: 10.1371/journal.pone.0220627 (PMC6675096; doi:10.1371/journal.pone.0220627)
Supplement: S5 File — Formulations tested in the internalization assay. (PPTX) [file pone.0220627.s005.pptx]

## Slide 1
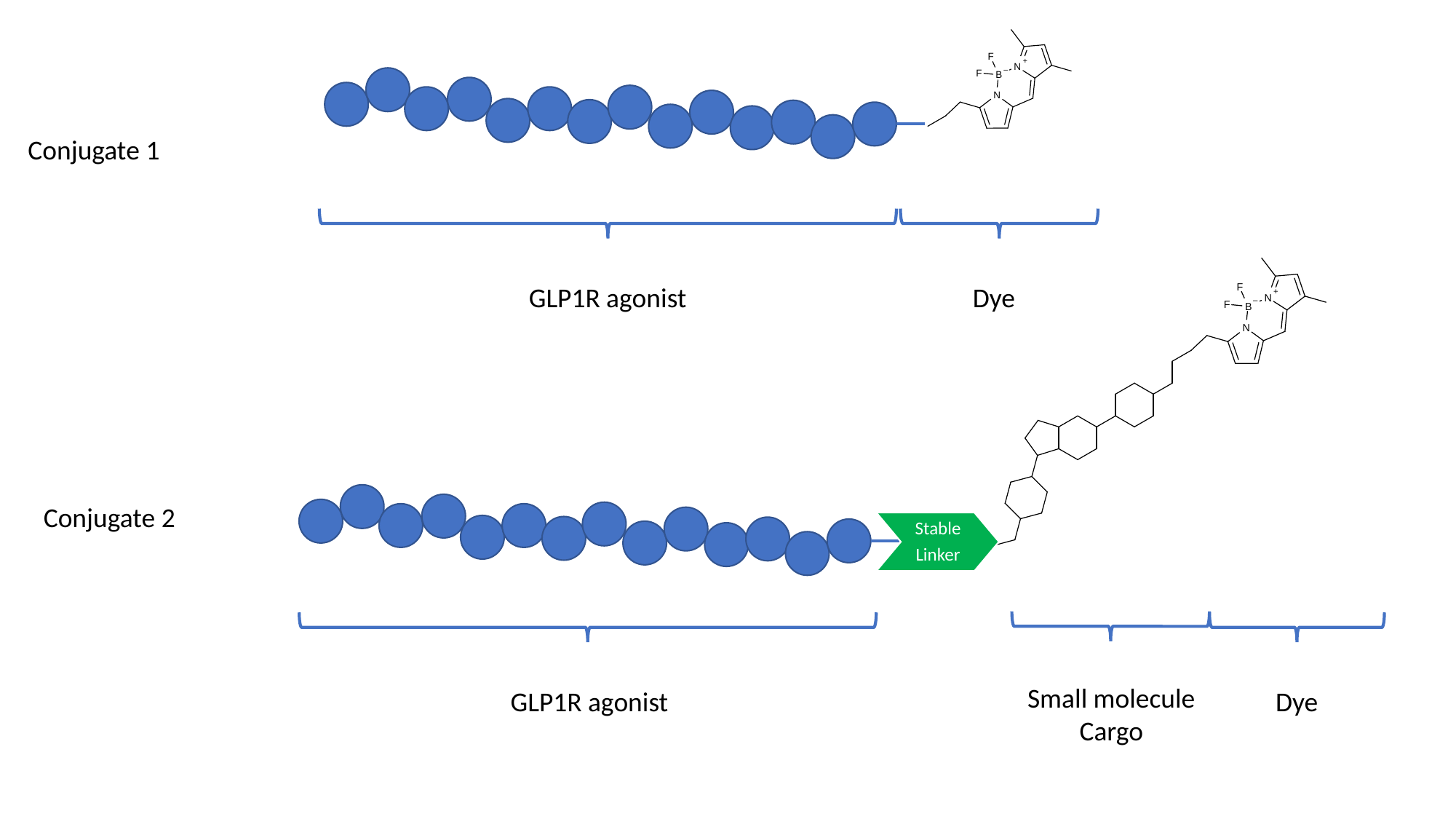

Conjugate 1
GLP1R agonist
Dye
Conjugate 2
Stable
Linker
Small molecule
Cargo
GLP1R agonist
Dye

## Slide 2
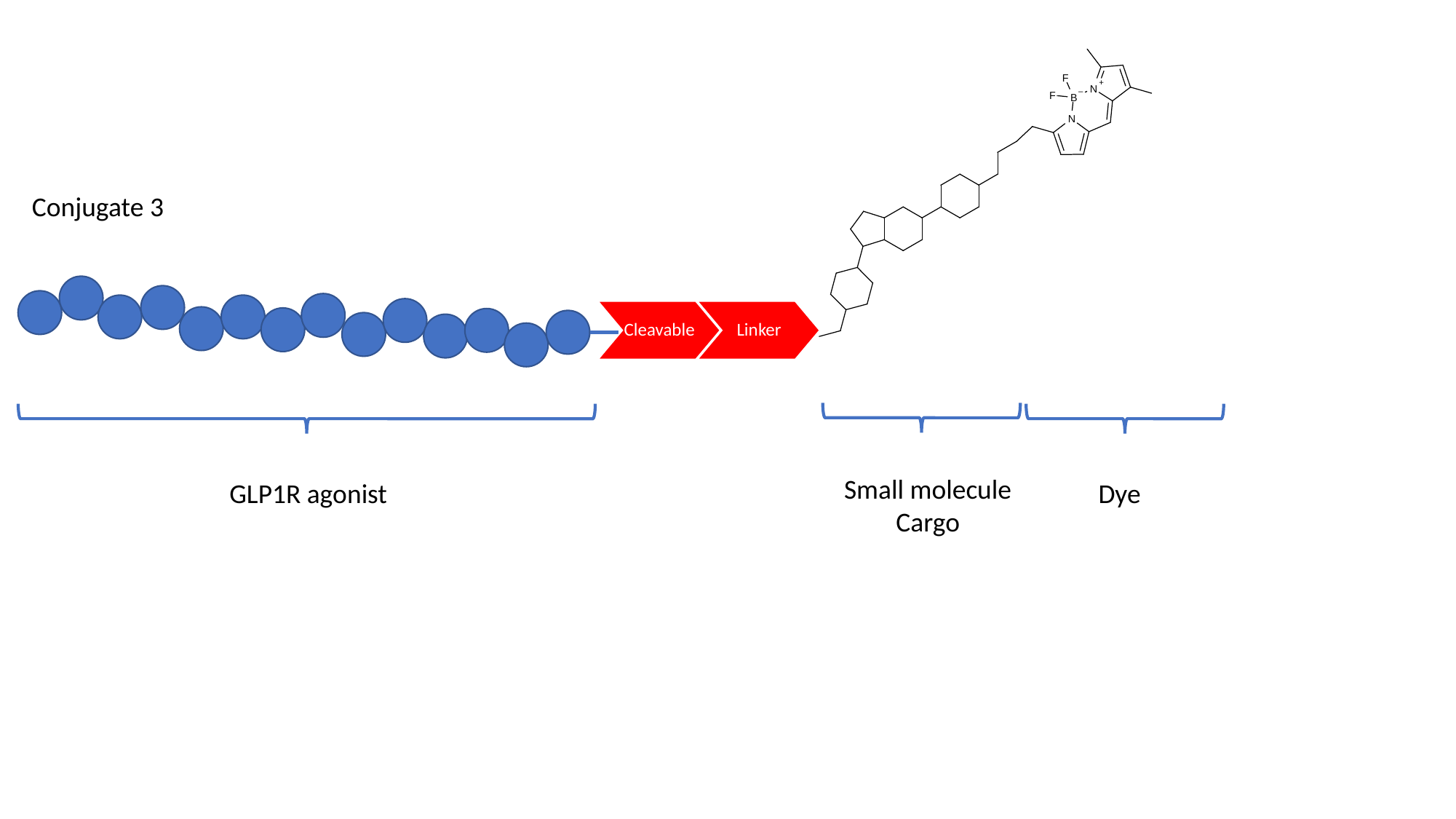

Conjugate 3
Cleavable
Linker
Small molecule
Cargo
GLP1R agonist
Dye

## Slide 3
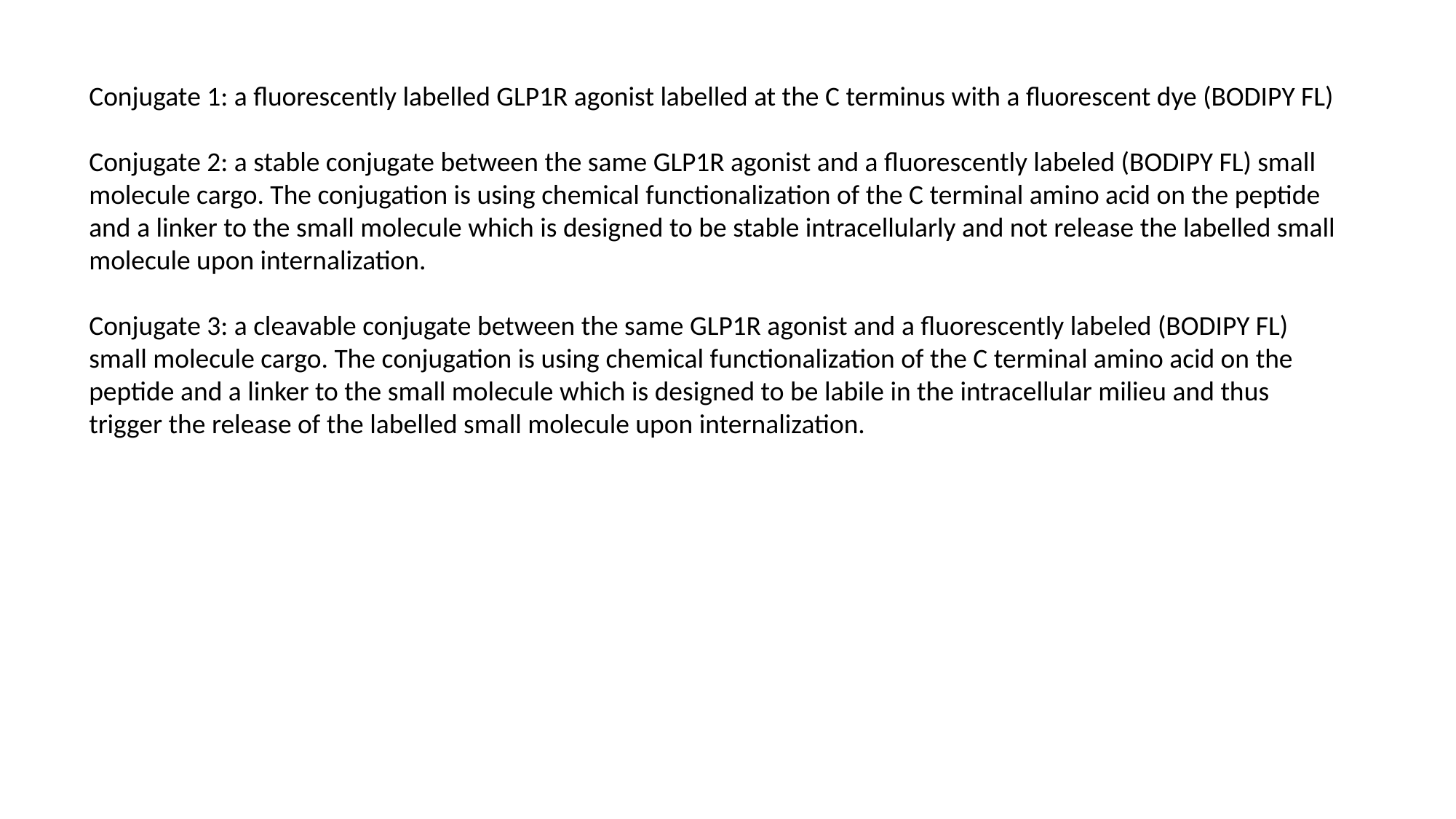

Conjugate 1: a fluorescently labelled GLP1R agonist labelled at the C terminus with a fluorescent dye (BODIPY FL)
Conjugate 2: a stable conjugate between the same GLP1R agonist and a fluorescently labeled (BODIPY FL) small molecule cargo. The conjugation is using chemical functionalization of the C terminal amino acid on the peptide and a linker to the small molecule which is designed to be stable intracellularly and not release the labelled small molecule upon internalization.
Conjugate 3: a cleavable conjugate between the same GLP1R agonist and a fluorescently labeled (BODIPY FL) small molecule cargo. The conjugation is using chemical functionalization of the C terminal amino acid on the peptide and a linker to the small molecule which is designed to be labile in the intracellular milieu and thus trigger the release of the labelled small molecule upon internalization.
